# Impact of socio-economic inequity in access to maternal health benefits in India: Evidence from *Janani Suraksha Yojana* using NFHS data

**DOI:** 10.1371/journal.pone.0247935

**Published:** 2021-03-11

**Authors:** Prem Shankar Mishra, Karthick Veerapandian, Prashant Kumar Choudhary

**Affiliations:** 1 PhD Research Scholar, Population Research Centre, Institute for Social and Economic Change, Bengaluru, Karnataka, India; 2 PhD Research Scholar, Center for Economic Studies and Policy, Institute for Social and Economic Change, Bengaluru, Karnataka, India; 3 PhD Research Scholar, Centre for Political Institutions, Governance and Development, Institute for Social and Economic Change, Bengaluru, Karnataka, India; Institute of Economic Growth, INDIA

## Abstract

**Background:**

Caste plays a significant role in Indian society and it influences women to health care access in the community. The implementation of the maternal health benefits scheme in India is biased due to caste identity. In this context, the paper investigates access to *Janani Suraksha Yojana* (JSY) among social groups to establish that caste still plays a pivotal role in Indian society. Also, this paper aims to quantify the discrimination against Scheduled Castes/Scheduled Tribes (SCs/STs) in accessing JSY.

**Methods:**

This paper uses a national-level data set of both NFHS-3 (2005–06) and NFHS-4 (2015–16). Both descriptive statistics and the Fairlie decomposition econometric model have been used to measure the explained and unexplained differences in access to JSY between SCs/STs and non-SCs/STs groups.

**Results:**

Overall, the total coverage of JSY in India is still, 36.4%. Further, it is found that 72% of access to JSY is explained by endowment variables. The remaining unexplained percentage (28%) indicates that there is caste discrimination (inequity associated social-discrimination) against SCs/STs in access to JSY. The highest difference (54%) between SCs/STs and non-SCs/STs in access to JSY comes from the wealth quintile, with the positive sign indicating that the gap between the two social groups is widening.

**Discussion and conclusion:**

It is necessary for the government to implement a better way to counter the caste-based discrimination in access to maternal health benefits scheme. In this regard, *ASHA* and *Anganwadi* workers must be trained to reduce the influence of dominant caste groups as well as they must be recruited from the same community to identify the right beneficiaries of JSY and in order to reduce inequity associated with social-discrimination.

## Background

One of the Sustainable Development Goals (SDG) is to reduce the global maternal mortality ratio (MMR) to less than 70 per 100,000 live births by 2030. Worldwide, about 295,000 maternal deaths were registered during 2017, of which most (approximately 86%) were reported from Sub-Saharan Africa and Southern Asia [1]. While Southern Asia accounts for nearly one-fifth of all maternal deaths, the region has registered the greatest overall reduction in MMR by nearly 60%, that is, from 384 to 157 deaths during pregnancy and childbirth [1]. However, the Indian side of the story is slightly different from that of the other Southern Asian countries. It is heartening that the MMR of India has declined from 167 in 2011–2013 to 130 in 2014–2016 and further, from 122 in 2015–17 to 113 in 2016–18. The MMR declined by 6.2% and 7.4% in the country during the periods 2014–2016 to 2015–2017 and 2015–17 to 2016–18 respectively, paving the way for the SDG target to be achieved much before the due year of 2030 [2]. As per the Maternal Mortality Bulletin, nearly 2,000 maternal deaths were averted per year during this period. This success story was made possible through the concerted efforts of the Indian government since the launch of the National Rural Health Mission (NRHM-2005) / National Health Mission (NHM-2013) [3]. Increased access to quality healthcare and wide coverage of health services under this programme have majorly contributed to the decline in MMR, infant mortality, and child mortality rates. Due to this push factor, the share of institutional deliveries, including in private facilities, rose to 79% in 2016 from 18% in 2005 [4,5].

Despite these achievements, the utilization of maternal healthcare services is still low in India [6]. This is due to sluggishness in healthcare progress, uneven distribution of healthcare services, and concentration of services in one particular place, region, and group [7,8]. Women belonging to poor, marginalized, and disadvantaged groups face greater hardships in access to healthcare services than those from rich, non-marginalized, and advantaged groups [9–14]. For instance, therefore (95%) of the economically well-off households receive institutional delivery services than the worse-off households (59%). In terms of social groups, 68%, 78%, and 80% of the women belonging to Scheduled Tribes (STs), Scheduled Castes (SCs), and Other Backward Classes (OBCs) respectively receive institutional delivery services, whereas as many as 83% women belonging to *Forward* Castes (FCs) receive those services. The availability of skilled birth attendants during pregnancy among women varies in the same pattern [4,15]. Further, the majority of *Forward* Castes and well-off households receive MCH services in both public and private institutions, however, lower castes groups and worse-off households depend mainly on public health institutions and receive lesser services due to multiple social and structural factors [16]. Therefore understanding these social and structural factors underlying MCH services required an assessment and also studying the distribution of healthcare benefits across socio-economic groups becomes imperative in the Indian context. A few studies have reported massive differences in access to healthcare benefits within and between socio-economic groups [17,18]. However, a dearth of literature was found in order to understand the inequity associated with socio-discrimination in maternal health benefits scheme in India. With this background, this paper examines the disadvantage suffered in receiving maternal health benefits by the SC and ST women as compared to their counterparts. The study also examines the determinants of access to maternal health benefits in India.

### Issues in access to maternal health benefits in India

In general, access to maternal health is influenced by many factors. These can be broadly grouped as supply-side and demand-side factors. Demand-side factors that influence inequity in the use of and access to maternal healthcare services are recognized as the socio-economic and contextual factors [7,16]. Further, these factors are also underlying with women’s social determinists of health in society [7,9,19,20]. However, from the supply-side, inadequacy of the institutional structure and the underlying systems in providing services is recognized as a barrier perceived by women [5]. Along with that, there is an enormous lack of political will regarding remedying the social health inequities that are highly prevalent in Indian society [14]. The tremendous inequity in access to maternal health care services among socio-economic groups is a major global concern [21–23]. To address this issue, the WHO report on ‘Commission on Social Determinants of Health’ in 2008, is provided a useful framework to deal with the issues of inequity and inequality prevail among marginalized and disadvantaged communities [21]. This health policy framework aims to reduce health inequity among the most vulnerable and marginalized groups across the world. In India, Scheduled Castes and Scheduled Tribes women are the most deprived and marginalized groups in seeking healthcare services compared to their counterparts [7,24,25].

Seeking institutional delivery by poor, disadvantaged, and rural pregnant women face multiple burdens [11,18,25]. The financial burden is one of the fundamental factors which restrict pregnant women from delivering their babies at healthcare institutions [10,11,26]. Women who deliver childbirth at health institutions bear out-of-pocket expenditure which ranges from arranging a vehicle to go to the hospital, spend on medicine, staying at the health center, and many more [11,26,27]. In India, still, after a huge investment in the public health system, the institutional delivery remains low across states, regions, and socio-economic groups [28–31]. Although achieving Universal Health Coverage (UHC) is one of the major SDGs that intends to provide financial risk protection to all eligible beneficiaries that are in need along with access to quality and essential healthcare services. Many low- and middle-income countries have introduced UHC programmes for the benefit of their citizens as a whole and particularly the poor and marginalized groups [32–35]. India too introduced a conditional cash transfer scheme called *Janani Suraksha Yojana* (JSY) in 2004 and launched in 2005 by special focusing on low-performing states to promote institutional delivery and post-natal care to reduce MMR and child mortality rates (CMR). This is one of the most extensive demand-side financing programmes launched in India [32]. It ensures safe delivery for all women aged 19 and above who belong to an SC/ST group and are below the poverty line (BPL) at the time of child delivery. ASHA (Accredited Social Health Activists) health workers play a significant role in ensuring community health by tracking women from pregnancy to childbirth and post-natal care. ASHA being a community health worker, remained a major catalyst for accelerating the institutional deliveries across the country. Each ASHA is engaged with the JSY scheme as a link between the government’s health system and poor pregnant women from the community. Studies have shown that financial assistance during pregnancy has led to greater utilization of maternal services [36–39]. However, all eligible beneficiaries do not receive the cash payment under the JSY scheme, and low coverage and under-utilization of the scheme have been found to prevail across the socio-economic groups [40,41]. Several studies have also reported that the practice of laying conditions for availing of the JSY cash transfer scheme during pregnancy prevents many eligible women from accessing JSY [5,34,39,41]. Therefore it becomes imperative to understand the low-coverage of JSY across the eligible beneficiaries among SC/ST women and the factors behind it.

As the Indian society is caste-based, the caste factor plays a significant role in all kinds of economic outcomes. Caste, as a predictor of economic outcomes, can also be correlated with occupation and employment [42–44], income and expenditure [45], capital [46], and access to credit [47]. As the healthcare system is considered as one of the economic outcomes, it is expected that caste can play a major role in healthcare access too. Studies such as those by Kulkarni and Baraik [48], Borooah et al. [8], and Acharya [19,20] have exposed that caste influences access to healthcare systems. And further, it has found a huge disparity in availing necessary maternal and child care services across social groups [7,19,25,26]. Social discrimination in health and healthcare practices puts women from poor and disadvantaged groups at high risk [7]. Other studies such as those by Nayar [49], Borooah [26], and Sabharwal [50] have also identified that lower caste women face discrimination in accessing essential reproductive healthcare services compared to higher caste women. To the best of our knowledge, no study has so far reported that caste discrimination prevails in access to the JSY cash payment scheme among the social groups in India. And, therefore, this study aims to understand the disparities and discrimination prevalent against the SC/ST women in Indian society as they seek to avail the JSY cash payment scheme. The analysis also seeks empirical evidence on how health policymakers can enhance the programme and revisit the implementation strategy.

## Materials and methods

**Fig 1:**
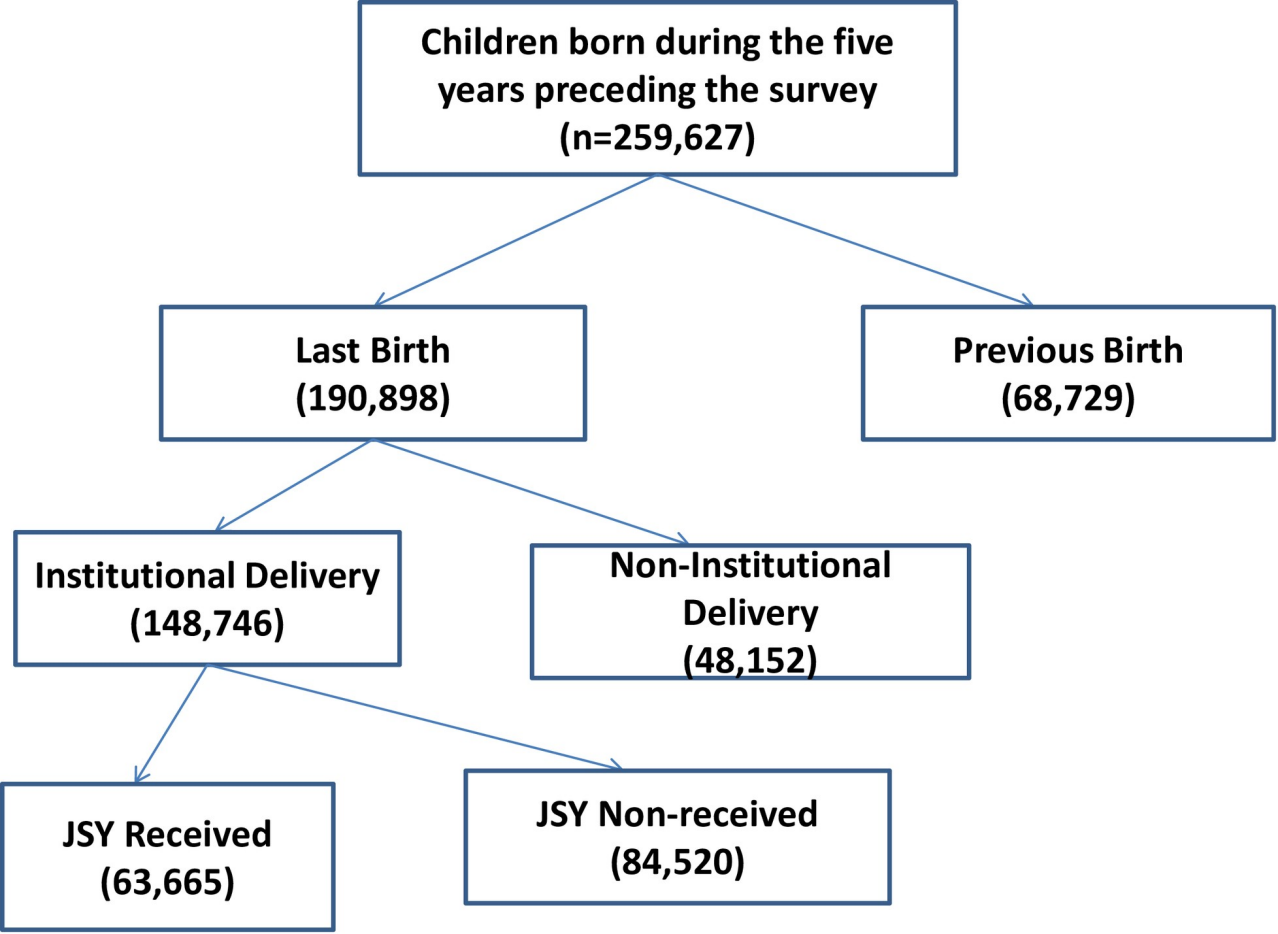
Schematic presentation of children born in five years preceding the survey by place of delivery and JSY beneficiaries in India, NFHS-4, 2015–16.

On the completion of ten years of the National Rural Health Mission (NRHM) [51], under which the JSY programme was launched to promote institutional delivery among the poor and marginalized members of the community, a need was felt to focus on the achievements of the JSY scheme and explore the scope for improvement. In order to study access to JSY among different social groups and states, we used unit-level data extracted from the National Family Health Survey (NFHS). The NFHS is a large-scale and multi-round survey conducted in a representative sample of households across India by the International Institute for Population Sciences (IIPS), Mumbai, since 1992–93. So far, four rounds have been conducted, the latest one having been surveyed during 2015–16 (NFHS-4). The NFHS provides vital data on health and family welfare and other issues related to them at both national and state levels. For the descriptive statistics, we used data from NFHS-3 [15] and NFHS-4 [4], where the sample sizes of women aged 15–49 years were 124,385, and 699,686 respectively. The detailed sampling design, coverage, and findings of the survey are available in the report [4]. To analyze the determinants of access to JSY and to decompose the access to JSY across social groups, the study used only NFHS-4. The samples used were the births that took place during five years preceding the survey, making the total sample size equal to 259,627. The unit-level data from the kids-file is used that covered 190898 births (last birth) in five years preceding the survey. Of these births, 148746 births were conducted in the health facilities of which JSY assistance was provided to the mothers of 63665 births. Fig 1 gives details about how the sample is drawn for the analysis. Further, the categorization of EAG (Empowered Action Group) states and Non-EAG states has followed the methodology of Sample Registration System, the office of registrar general of India, which publishes Special Bulletin on MMR [2]. The Maternal Mortality Bulletin (2014–2016) categorizes the Indian states as Empowered Action Group plus (EAG plus), Southern states, and Others in order to better understand MMR and its distributional regional patterns. The EAG plus states are Bihar, Jharkhand, Madhya Pradesh, Chhattisgarh, Odisha, Rajasthan, Uttar Pradesh, Uttarakhand, and Assam, while the southern states are Andhra Pradesh, Telangana, Karnataka, Kerala, and Tamil Nadu. The remaining states/UTs are categorized as ‘others.

### Ethics statement

The data used in the study is available in the public domain with no identifiable information on the survey participants; therefore, no ethics statement is required for this work. The National Family Health Survey (NFHS) is the largest health survey in the world, which is conducted by the International Institute for Population Sciences (IIPS), Mumbai, under the Ministry of Health and Family Welfare, Government of India. The first survey was conducted in 1992. The 2^nd^ and the 3^rd^ rounds were conducted in 1998 and 2005–06 respectively. The 4^th^ and latest round was conducted during 2015–16.

### Econometric methodology

The decomposition method was first developed by Blinder [52] and Oaxaca [53] to study the discrimination between males and females in the labor market. Later, the application was extended to and applied in other sectors to compare privileged (advantaged) and non-privileged (disadvantaged) groups. Most studies apply the linear model to decompose the outcome variable between advantaged and disadvantaged groups. Since access to JSY is usually not random but based on specific socio-economic characteristics, the dependent variable is binary (access to JSY = 1, otherwise = 0). Hitherto, as per our knowledge, there have been no studies that have decomposed JSY as a health outcome. Our study is the first one to decompose the access to JSY into explained and unexplained differences between two groups using the non-linear Fairlie decomposition model [54]. For the analysis, we combined both SCs and STs and referred to them as SC/ST and referred to the rest of the caste groups (both OBCs and FCs) as non-SC/ST. As mentioned in the Fairlie decomposition model [54], the gap in the average value of the dependent variables between non-SCs/STs and SCs/STs, $i.e.{\overset{´}{Y}}^{n}-{\overset{´}{Y}}^{s}$ (i.e. access to JSY) can be written as:

$${\overset{´}{Y}}^{n}-{\overset{´}{Y}}^{s}=[({\overset{´}{X}}^{n}-{\overset{´}{X}}^{s}){\hat{\beta }}^{n}]+[{\overset{´}{X}}^{s}({\hat{\beta }}^{n}-{\hat{\beta }}^{s})]$$

Unlike the Blinder-Oaxaca [52,53] model, the Fairlie decomposition [54] follows the logistic distribution function because of the non-linear binary (1, 0) dependent variables. In the above equation, the first term in brackets denotes explained differences, that is, the gap between the two groups due to group differences in the distribution of the endowment variables. The second term represents unexplained differences that capture the gap due to group differences in unmeasurable or unobserved endowments (see detailed Bora et al., [55] for further understanding of Fairlie decomposition). Moreover, the descriptive analysis of the variables used in the study can be found in the supporting document (supporting file 1)

## Results

### Status of childbirth and place of delivery in India

Since the commencement of NRHM and JSY in 2005, there has been a reduction of in-home delivery while substantially increased childbirth at public and private hospitals. JSY is mainly meant to encourage women to deliver in public hospitals. In this regard, the country has shown improvement in the last decade as the share of public hospital delivery increased from 18 to 52% while that of home delivery declined significantly from 61 to 21% from 2005 to 2015 (Table 1). Regarding childbirth at home, the southern states did a better job by bringing their share of home delivery down to 8%, which is lower than that of EAG plus states (29%) and other states (13%). This suggests that more healthcare facilities are available in the southern states. But whether they are affordable for everyone is a vital question that has to be answered. In the case of childbirth in public hospitals, all the states performed equally well, taking their share to above 50% in 2015. The share of childbirth in private hospitals also increased at the national level by 6% between 2005 and 2015. This suggests that there is a need to revamp the policies to empower public hospitals to reduce MMR due to home delivery as well as reduce exploitation by private hospitals. Furthermore, a detailed analysis can be found in the supporting document (supporting file 2).

**Table 1:** Distribution of childbirth by place of delivery in 2005 and 2015 (%).

Sl. No	State groups	Home Delivery		Public Hospitals		Private Hospitals	
		2005	2015	2005	2015	2005	2015
1.	EAG states	76.9	28.5	10.3	53.2	12.8	18.2
2.	Southern states	25.8	7.6	34.3	50.4	39.9	42.0
3.	Other states	48.4	13.2	24.8	50.9	26.7	35.9
	All India	61.1	20.8	18.0	52.1	20.8	27.1

Source: Author’s calculation based on NFHS-3 (2005–06) and NFHS 4 (2015–16).

**Fig 2:**
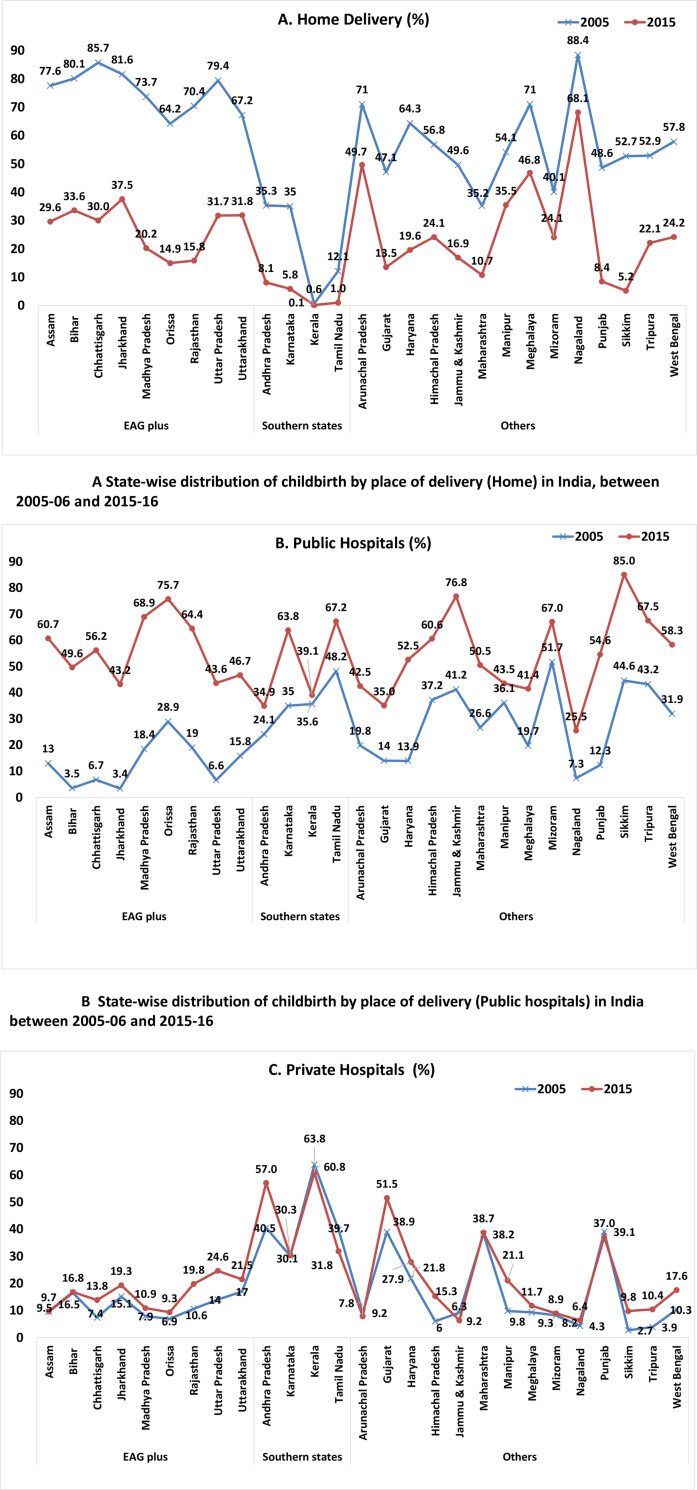
A, B & C. State-wise distribution of childbirth by place of delivery in India.

Major state-wise distribution of childbirth shows that the share of childbirth at home declined in almost every state during 2005–15 (*Fig 2A*). Kerala recorded the least number of childbirths at home, while Nagaland topped the list with over 60% of the childbirths taking place at home in 2015. Among the EAG plus states, Orissa recorded the least (15%) share of home delivery, whereas Jharkhand recorded the highest (38%) share in 2015. Compared with the other two groups, EAG plus states had more childbirths at home. In the case of public hospital delivery, except the EAG states of Jharkhand, Uttar Pradesh, and Uttarakhand, the other EAG states exhibited better results with more than 50% public hospital deliveries in 2015 (*Fig 2B*). Among the southern states, Tamil Nadu recorded the most (67%) number of childbirths in public hospitals, followed by Karnataka (64%). Among all the states, Sikkim recorded the highest (85%) number of childbirths in public hospitals and Nagaland the lowest (7%). In a few states, both childbirth at home and public hospitals show contrary results. For instance, Nagaland recorded the least number of births in hospitals whether private or public, whereas Kerala recorded the most number of private hospital deliveries. These results strongly suggest that these states have to improve public health facilities.

Women from Kerala, Andhra Pradesh, and Gujarat prefer private hospitals for child delivery (*Fig 2C*). Though the number of childbirths in public hospitals has increased in the last decade, the share of private hospitals is also very high in some states. Overall, improvement has been undoubtedly witnessed in institutional delivery whether in public or private hospitals, and it has been possible only through the implementation of the demand-side financing programme to empower all pregnant women.

**Fig 3:**
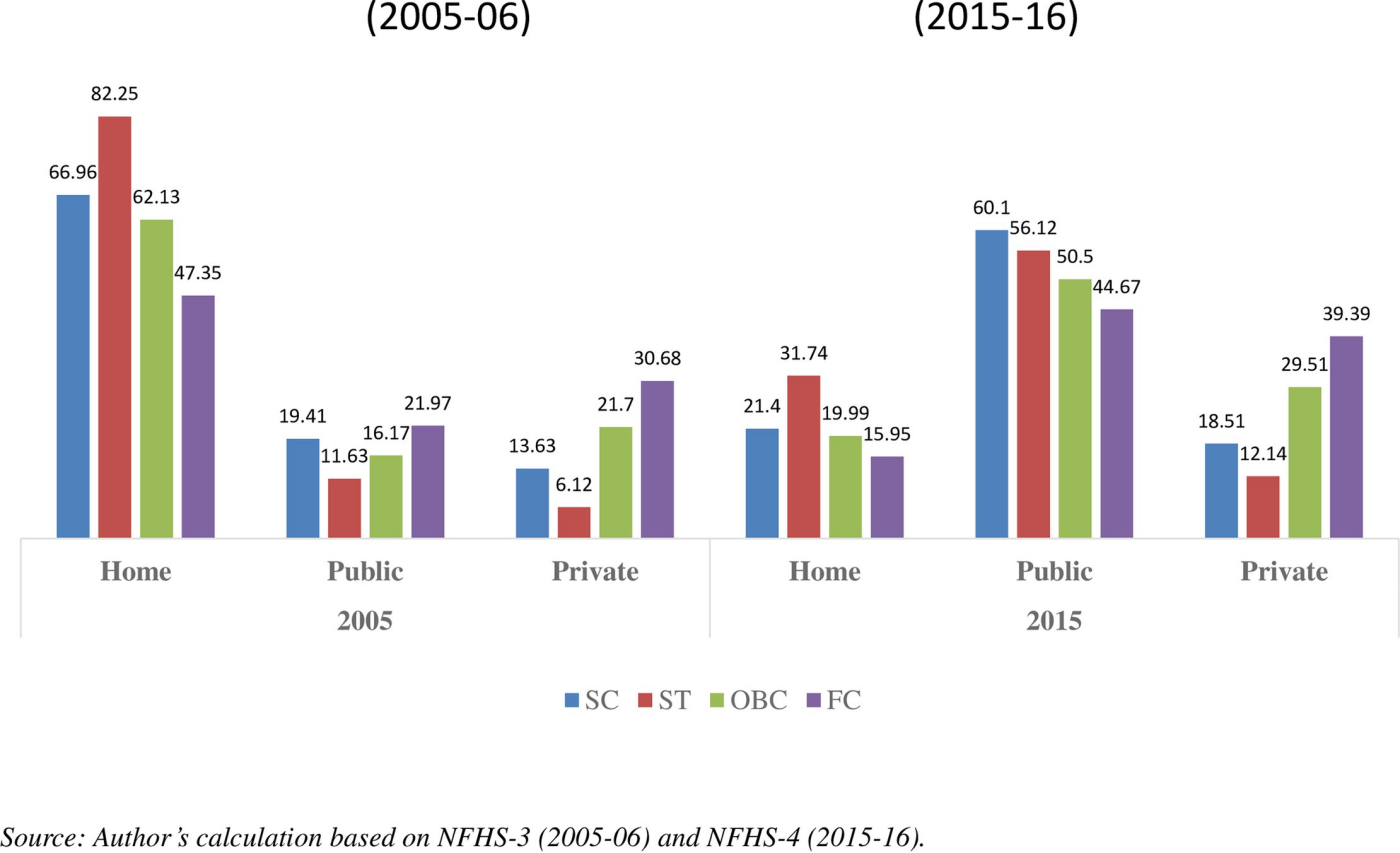
Distribution of institutional delivery (%) across social groups in India.

However, it is necessary to understand whether all women, irrespective of caste, religion, and other related circumstances, can access JSY. From *Fig 3*, it is clearly visible that there were widespread persistent disparities in childbirth at home and public and private institutions among social groups between 2005 and 2015. During this period, deliveries at home reduced drastically. The rate of public delivery increased manifold across the groups, and the rate of private hospital delivery also increased to some extent among various social groups. Yet, differences in access to JSY exist among social groups. Home delivery among SCs/STs is higher than among non-SCs/STs, whereas private hospital delivery is lower. Childbirth in public hospitals is high among SCs/STs and that has given them access to JSY financial assistance. However, a few studies are sceptical and say that these differences in access to JSY are, in fact, due to caste practices in India [34,41].

### Access to JSY among social groups

Women in India who are poor, marginalized, and live in remote areas face financial hardships in access to healthcare institutions, which in turn leads to high levels of MMR. In order to address this, JSY, a cash transfer programme aimed at reducing MMR and facilitating childbirth at public hospitals, was introduced in 2005. A decade after its inception, its national coverage stood at 36.4% in 2015–16 (Table 2). The eligibility criteria for pregnant women to avail of JSY vary across low and high performing states in India is followed as; in the low-performance states, all pregnant women who undergo childbirth at the public or accredited private institutions are eligible to receive assistance under the scheme, whereas in the high-performance states, all pregnant women who belong to BPL households, belonging to SC/ST, are 19 years or above of age, and undergo childbirth at public or accredited private health institutions up to second births, are eligible.

**Table 2:** Share of access to JSY among social groups by major states during 2015-16(%).

Sl. No.	States	SC/ST	Non-SC/ST	Total
***I***	***EAG Plus Assam states***	***36*.*8***	***62*.*6***	***57*.*3***
1.	Uttar Pradesh	30.1	69.8	48.7
2.	Bihar	26.4	73.4	53.9
3.	Uttarakhand	28.9	69.6	49.4
4.	Chhattisgarh	47.6	52.3	66.2
5.	Jharkhand	39.9	59.9	41.6
6.	Madhya Pradesh	38.5	61.1	61.1
7.	Rajasthan	37.5	61.5	56.1
8.	Orissa	48.3	51.1	72.6
9.	Assam	34.0	65.1	66.1
***II***	***Southern states***	***35*.*6***	***63*.*7***	***19*.*8***
10.	Andhra Pradesh	39.8	59.9	17.4
11.	Telangana	35.7	62.7	11.9
12.	Karnataka	42.7	56.1	19.9
13.	Tamil Nadu	37.9	62.1	29.5
14.	Kerala	22.1	77.6	20.4
***III***	***Other states***	***62*.*5***	***36*.*7***	***25*.*7***
15.	Arunachal Pradesh	76.0	23.4	20.5
16.	Gujarat	47.7	49.7	8.9
17.	Haryana	54.1	45.7	13.5
18.	Himachal Pradesh	50.9	48.8	13.1
19.	Jammu and Kashmir	39.6	60.1	54.0
20.	Maharashtra	53.9	45.8	8.7
21.	Manipur	31.2	67.0	26.2
22.	Meghalaya	99.6	0.4	28.0
23.	Mizoram	99.1	0.3	47.5
24.	Nagaland	98.7	1.3	29.4
25.	Punjab	58.3	41.7	19.1
26.	Sikkim	44.7	54.9	29.4
27.	Tripura	67.9	31.7	32.6
28.	West Bengal	53.2	43.6	28.7
	***All India***	***37*.*6***	***61*.*8***	***36*.*4***

Source: Author’s calculation based on NFHS-4 (2015–16).

The national average of access to JSY is 36%, and only a few states such as all EAG plus Assam states (above 41%), Mizoram (48%), and Jammu & Kashmir (54%) have crossed this average (Table 2). With 72.6% access to JSY, Orissa stands at the top, while with 8.7 and 8.9% access respectively, Maharashtra and Gujarat are the bottoms. Since India faces an immense health inequity due to persisting socio-economic inequality, social discrimination in access to JSY is also associated with low access to and under-utilization of healthcare services across states. At the social group level, the national share of SCs/STs in access to JSY is lower (38%) than that of non-SCs/STs (62%). Naturally, there is a variation at the state level in the access to JSY between SCs/STs and non-SCs/STs. Non-SCs/STs have a greater share than their SC/ST counterparts in both the EAG plus and the southern states in the total access to JSY. Arunachal Pradesh, Haryana, Himachal Pradesh, Maharashtra, Meghalaya, Mizoram, Nagaland, Punjab, Tripura, and West Bengal are the major states in which the access of SCs/STs to JSY is higher. SCs/STs in the rest of the states have lower access to JSY than non-SC/STs. In many states, there is a contradiction between the number of childbirths in public hospitals and the access of SCs/STs to JSY. In other words, states childbirth in public hospitals among SCs/STs is higher show less access to these social groups to JSY. This contradiction clearly shows that discrimination prevails in access to JSY against SCs/STs.

### Determinants of access to JSY by caste

From the above descriptive results, it is evident that access to JSY depends on caste. To further show that caste influences access to JSY, we applied the Fairlie decomposition method, followed by the Blinder-Oaxaca [52,53] model. First, the model estimated the augmented logistic regression function separately for SCs/STs and non-SCs/STs. The dependent variables were dichotomous (1 = access to JSY, 0 = otherwise), while the group of independent variables was qualitative viz., delivery type, birth order type, level of woman’s education, employment and age, level of husband’s education and employment, quintile of household wealth, religion, and residential place (rural or urban). The rationale behind selecting these independent variables is that JSY is an economic welfare scheme to help pregnant women during childbirth and may be affected by socio, economic, demographic, and geographic variables. Since this scheme promotes institutional delivery, it is meant for childbirth in public hospitals irrespective of whether it is a caesarean birth or a normal delivery. But the negative coefficients of both SC/ST and non-SC/ST women show that as compared to women having a normal delivery, those having a caesarean birth delivery have less likelihood of access to JSY (Table 3). The positive coefficients of the birth order, irrespective of caste, show that the delivery of the second and each successive child has more likelihood of access to JSY.

**Table 3:** Determinants of access to JSY: *Logistic regression results*.

Sl. No.	Variables Name	SC/ST		Non-SC/ST	
		Coefficients	Standard Error	Coefficients	Standard Error
1.	**Type of birth** *(Ref*: *normal birth)*				
	Caesarean birth	-0.852***	(0.028)	-1.066***	(0.021)
2.	**Birth order of the child** *(Ref*: *1*^*st*^ *child)*				
	2 and more	0.0009	(0.021)	0.062***	(0.017)
3.	**Age-group of the woman** *(Ref*: *above 30)*				
	Age 15–17	-0.793***	(0.143)	-0.815***	(0.138)
	Age 18–30	-0.146***	(0.020)	-0.029**	(0.016)
4.	**Education of the woman***(Ref*: *illiterate)*				
	Primary	-0.029	(0.029)	0.014	(0.026)
	Secondary	-0.025	(0.024)	-0.109***	(0.021)
	Higher secondary and above	-0.022***	(0.044)	-0.453***	(0.031)
5.	**Occupation of the woman***(Ref*: *organized sector)*				
	Unemployed	-0.102*	(0.057)	-0.051*	(0.037)
	Unorganised	0.019	(0.071)	-0.061	(0.057)
6.	**Occupation of the Husband** *(Ref*: *organized sector)*				
	Unemployed	-0.269**	(0.114)	-0.088	(0.091)
	Agriculture	0.059	(0.067)	-0.055	(0.050)
	Unorganised	0.169***	(0.064)	0.068	(0.044)
7.	**Wealth quintile** *(Ref*: *rich)*				
	Poor	0.729***g	(0.029)	0.867***	(0.022)
	Middle	0.333***	(0.028)	0.507***	(0.020)
8.	**Religion** *(Ref*: *others)*				
	Hindu	0.448***	(0.022)	0.279***	(0.019)
9.	**Place of residence** *(Ref*: *urban)*				
	Rural	0.174***	(0.025)	0.303***	(0.018)
10.	**Media**				
	Radio	0.039	(0.026)	0.049***	(0.021)
	TV	-0.049**	(0.022)	-0.066***	(0.019)
	Newspaper	-0.073***	(0.028)	-0.209***	(0.020)
	Constant	-0.718***	(0.044)	-0.904***	(0.034)
	Log likelihood	- 33775.728		-53559.386	
	Samples (N)	51,896		89,195	

Source: Author’s calculation based on NFHS-4 (2015–16).

Note: The dependent variable is access to JSY.

***p < 0.01

**p < 0.05

*p < 0.1.

Women belonging to the age group above 30 years have more likelihood of access to JSY than women less than 30 years of age. One significant observation is that women in the age group of 15–17 years have a very less likelihood of access to JSY. Since marriage in this age group is legally considered as child marriage, pregnant women in this age group likely do not seek the benefit of JSY. The coefficients of the educational level of women are negative for both SCs/STs and non-SCs/STs, which shows that education does not influence access to JSY. Despite the negatively significant coefficient of the higher education in the case of both SCs/STs and non-SCs/STs, the likelihood of access to JSY among SCs/STs increases only when they have higher secondary and above levels of education. This result highlights that either non-SC/ST pregnant women do not seek JSY as most of them prefer private hospitals over public hospitals or that they do not need government hospital facilities. Compared to women working in the organized sector, the likelihood of access to JSY among unemployed women is less among both SCs/STs and non-SCs/STs. This underscores the need for the JSY policy to be revamped for the benefit of unemployed women too. The coefficients of occupation of the husband are seen to increase positively, irrespective of significance, as we go from the unemployed sector to the unorganized sector. This shows that husbands from both SCs/STs and non-SCs/STs are well aware of JSY due to their peer group workers.

When it comes to economic factors, wealth quintile is the most important factor determining access to JSY. The result shows that women from middle and poor quintiles have higher chances of accessing JSY than those from the rich quintile. This is possibly due to poor women’s preference for government hospitals due to the belief that they are easily available, affordable, and good. In the case of religion, as the majority of the women are Hindus, they have more access to JSY than the non-Hindus. This indicates that women from minority religions are deprived of access to JSY. Rural women are more likely to access JSY than urban women irrespective of caste. However, the higher (0.303) likelihood ratio of rural non-SC/ST women shows that they have more access to JSY than their SC/ST counterparts (0.174). Access to JSY is possibly influenced by media contact as most welfare schemes are publicized in the media for the benefit of the public. In the case of radio, the coefficients are positive and significant for non-SCs/STs but not for SCs/STs. Both TV and newspapers have failed to influence households in access to JSY. This result strongly suggests the need for welfare schemes to be advertised through TV and newspapers as they are the most important sources of information. From the above logistic regression results, it is clear that access to JSY depends on many socio-economic and other variables. Like with descriptive statistics, the regression results also show that there are differences in access to JSY between SCs/STs and non-SCs/STs.

### Decomposition of access to JSY by caste

The Fairlie decomposition results are presented in Table 4. The results indicate that even after considering important variables, only up to 72% of access to JSY can be explained. This implies that there are still some other factors that influence access to JSY. As both Blinder-Oaxaca (10, 35) and Fairlie (17) elucidate, the remaining unexplained percentage (28%) is considered as discrimination coefficients due to the group differences between non-SC/STs and SC/STs.

**Table 4:** Differences in JSY access between SC/ST and non-SC/ST.

Sl. No.	Covariates	Access to JSY	
		Coefficient	Percentages to the total (%)
**Covariates contributions**			
1.	Caesarian birth	-0.015***	22.2
2.	Birth order of the child	-0.0002***	0.4
3.	Age-group of the women	-0.0001**	0.07
4.	Education of the women	-0.009***	12.5
5.	Occupation of the women	-0.001	2.0
6.	Occupation of the Husband	0.002	-2.2
7.	Wealth quintile	-0.038***	54.1
8.	Religion	0.003***	-4.5
9.	Place of residence	-0.007***	10.0
10.	Media	-0.004***	5.4
	**Total (= 1 to 10)**	**-0.069**	**100**
**Decomposition results**			
1.	Total explained gap	-0.069	71.9
2.	Total unexplained gap	-0.027	28.1
	**Total raw differentials (= 1+2)**	**-0.096**	**100**
Mean prediction of Non-SC/ST		0.392	
Mean prediction of SC/ST		0.489	
Samples (N)		1,41,091	

Source: Author’s calculation based on NFHS-4 (2015–16).

Note

***p < 0.01

**p < 0.05

*p < 0.1.

The contribution effect of each endowment shows that the highest difference in access to JSY between SCs/STs and non-SCs/STs is explained by the wealth quintile. The positive sign indicates that the gap between these two groups is to the extent of 54% (Table 4). The next disadvantage to SC/ST households comes from a caesarean birth, where there is a 22% gap between the two groups in access to JSY. This indicates that women from non-SC/ST households have more advantages than their SC/ST counterparts for child delivery. The educational level of women is also important in access to JSY as there is a 12.5% gap between the two groups. Even though most factors make a meager contribution individually, the fact that the variables are positive shows that access to JSY is significantly inclined towards non-SC/STs. Thus, it is very evident that both SC and ST women face discrimination in access to JSY due to the evil caste hierarchy in India just like in other economic outcomes such as access to a job, access to credit, and access to health services.

## Discussion and conclusion

JSY is a cash transfer programme that aims to reduce MMR and CMR during childbirth by promoting institutional delivery and post-natal care. This paper shows that access to JSY varies across states and social groups and there is a huge gap in access to the scheme even among the beneficiary groups. The all-India coverage of JSY is 36.4%. Except in a few states like Orissa, Chhattisgarh, Assam, and Madhya Pradesh, where access to JSY is above 60%, access in the remaining states like Maharashtra, Gujarat, and Telangana is less than 36%. A huge gap in the JSY coverage is clearly seen across the states with north-south divides. Similarly, in the social groups, the utilization of JSY services varied and it was found that the beneficiaries group is still lacking to access it. The difference in availing JSY services between SC/ST (37.6%) and Non-SC/ST (61.8%) women are 24.2 points. Likewise, the institutional delivery is shown vast differences between the pre-NRHM and post-NRHM periods across social groups, states, and regions. Even though a few states have a robust institutional delivery system, still, they do poorly in terms of access to JSY among eligible beneficiaries. While the scheme is expected to increase equity in the utilization of maternal healthcare services such as prenatal, natal, and post-natal care, the results revealed that there are inequalities and inequity associated with social-discrimination in the distribution of JSY across social groups that eventually lead to under-utilization of services too.

Though the scheme was initially to launch in the low performing states like EAGs plus Assam where the MCH service coverage was poor, later, it extended to all states and union territories. Due to the JSY scheme, the MCH service utilization among marginalized and poor women has increased [39,40]. However, the coverage of JSY services still remained the same, and the difference found between the social groups like SC/ST and Non-SC/ST is huge.

The paper finds that access to JSY is influenced by individual, household, and community-level factors. Social, economic, and demographic factors also determine access to JSY among social groups in the country [40]. Most of the factors such as birth order of the children, age and education of the women, occupation of the husband, household’s wealth status, place of residence, and media for spreading awareness plays a role in the under-utilization of and low access to the programme. The Fairlie decomposition analysis [54] shows that a wide gap of around 54% exists between SC/ST and non-SC/ST households due to differences in wealth. It shows that access to JSY is highly influenced by economic status. Both descriptive and econometric analyses conclude that caste plays a major role in access to JSY in India. Due to the caste hierarchy and the resultant discrimination, SC/ST women experience differential treatment at childbirth, leading to less economic and social well-being in the country [56]. Further, the mother’s education has also contributed to a 12.5% gap between SC/ST and Non-SC/ST women in influencing to access the JSY services.

Certainly, programmes and policy interventions related to JSY have made an impact on service accessibility [37], but social identity plays a role in maintaining the inequality of access and utilization of the services [5]. However, earlier studies have clearly revealed that there exist huge differential and discrimination across social groups in accessing healthcare services in the community [24]. Here, this study also provides some plausible explanations for the above findings with quantifiable evidence. Our findings revealed that there is inequity associated with social-discrimination in receiving JSY services across social groups that could be seen with the bivariate and multivariate analysis. The JSY programme was aimed to reduce the inequity and inequality in MCH care services across class, caste, rural-urban, and state-regions; however, the health system level barriers and the social determinants of health that still, constraints in reducing the gap which prevails in accessing social welfare scheme [5,34,57].

Furthermore, our findings also revealed that demand-side financing needs a proper implementation strategy that is apparently perceived in caste-wise institutional delivery in India. Thus, strengthening the health system is required in order to improve the supply-side mechanisms and it is also needed to enhance the interpersonal communications with community health workers and stakeholders that is lacking in the public health system in India [57]. This may have the ultimate effect on covering the existing programme to everyone.

In conclusion, there is needed to rectify all those issues, and to maintain the balance between access to and utilization of the scheme, our study has policy suggestions. The first is that since ASHA workers play a vital role in dealing with the community level issues from tracking pregnancy to childbirth, they have to be trained well to identify and avoid caste influences in access to JSY. Furthermore, both ASHA and *Anganwadi* workers must be encouraged to work together to identify the right beneficiaries of JSY. They must be trained to reduce the influence of dominant caste groups as well as they must be recruited from the same community in order to reduce inequity associated with social-discrimination. Finally, there needs to be a system of checks and balances in place to ensure that such problems do not arise in the first place. Only when measures like these are put in place can programmes like JSY succeed in providing services to all women without any biases and discrepancies. The JSY programme has a great influence on those women who belong to a disadvantaged community in utilizing more MCH services and therefore it should be promoted at every individual level with a universal approach.

## Supporting information

S1 File Descriptive statistics of main variables used in the OLS equation of access to JSY. (DOCX)

S2 File Percentage distribution of childbirth by place of delivery in major states of India and the differences between 2005–06 and 2015–16. (DOCX)

S3 File (DOCX)

## References

[1] WHO. (2019, September 19). Retrieved from World Health Organaization: https://www.who.int/news-room/fact-sheets/detail/maternal-mortality. 2019 Sept.

[2] *Special Bulletin on Maternal Mortality in India 2015–17*. New Delhi: *Sample Registration System, Office of Registrar General*, Vital Statistics Division, India 2019.

[3] UNICEF, India. Coverage Evaluation Survey (CES), 2009: All India report. New Delhi. 2010.

[4] IIPS, ICF. National Family Health Survey (NFHS-4), 2015–16: India. Mumbai: International Institute for Population Sciences 2017.

[5] Gupta A, Fledderjohann J, Reddy H, Raman VR, Stuckler D, Vellakkal S. Barriers and prospects of India’s conditional cash transfer program to promote institutional delivery care: a qualitative analysis of the supply-side perspectives. *BMC health services research*. 2018 12;18(1):40. 10.1186/s12913-018-2849-8 29370798 PMC5785836

[6] Paul S, Paul S, James KS. Universalisation versus targeting in maternal and child health care provisioning: Evidence from India. *SSM-population health*. 2019 12 1;9:100502. 10.1016/j.ssmph.2019.100502 31720361 PMC6838526

[7] Baru R, Acharya A, Acharya S, Kumar AS, Nagaraj K. Inequities in access to health services in India: caste, class and region. *Economic and Political Weekly*. 2010 9 18:49–58.

[8] Borooah VK, Sabharwal NS, Thorat S. Gender and caste-based inequality in health outcomes in India. Indian Institute of Dalit Studies; New-Delhi (*Working Paper Series 7(3)*) 2012.

[9] Blas E, Gilson L, Kelly MP, Labonté R, Lapitan J, Muntaner C, et al. Addressing social determinants of health inequities: what can the state and civil society do?. *The Lancet*. 2008 11 8;372(9650):1684–9. 10.1016/S0140-6736(08)61693-1 18994667

[10] Gwatkin DR. Health inequalities and the health of the poor: what do we know? What can we do?. *Bulletin of the world health organization*. 2000;78:3–18. 10686729 PMC2560590

[11] Houweling TA, Ronsmans C, Campbell OM, Kunst AE. Huge poor-rich inequalities in maternity care: an international comparative study of maternity and child care in developing countries. *Bulletin of the World Health Organization*. 2007;85:745–54. 10.2471/blt.06.038588 18038055 PMC2636501

[12] Jarris P, Savage-Narva Y, Lupi MV. Promoting health equity and optimal health for all. *Journal of Public Health Management and Practice*, 2016. 22, S5–S7. 10.1097/PHH.0000000000000377 26599029

[13] Marmot M, Allen JJ. Social determinants of health equity. 2014:104 Suppl4, S517–S519. 10.2105/AJPH.2014.302200 25100411 PMC4151898

[14] Navarro V, Muntaner C, Borrell C, Benach J, Quiroga Á, Rodríguez-Sanz M, et al. Politics and health outcomes. *The Lancet*. 2006 9 16;368(9540):1033–7. 10.1016/S0140-6736(06)69341-0 16980120

[15] IIPS, ORC-Macro. National Family Health Survey (NFHS-3), 2005–2006: India. Mumbai: International Institute for Population Sciences 2007.

[16] Sanneving L, Trygg N, Saxena D, Mavalankar D, Thomsen S. Inequity in India: the case of maternal and reproductive health. Global health action. 2013 12 1;6(1):19145. 1–31. 10.3402/gha.v6i0.19145 23561028 PMC3617912

[17] Devarajan S, Shah S. Making services work for India’s poor. *Economic and Political Weekly*. 2004 2 28:907–19.

[18] Jeffery P, Jeffery R. Only when the boat has started sinking: a maternal death in rural north India. *Social Science & Medicine*. 2010 11 1;71(10):1711–8. 10.1016/j.socscimed.2010.05.002 20561728 PMC2981873

[19] Acharya S. Caste and patterns of discrimination in rural public health care services. Blocked by Caste: Economic Discrimination in Modern India (New Delhi: Oxford University Press, 2010). 2010:208–29.

[20] Acharya SS. Conceptualizing social discrimination in access to health services towards measurement-illustrating evidences from Villages in Western India. *Indian Emergency Journal*. 2011;6(2):14–27.

[21] WHO Commission on Social Determinants of Health, World Health Organization. Closing the gap in a generation: Health equity through action on the social determinants of health: Commission on Social Determinants of Health final report. World Health Organization; 2008.

[22] Marmot M. Social determinants of health inequalities. *The Lancet*. 2005 3 19;365(9464):1099–104. 10.1016/S0140-6736(05)71146-6 15781105

[23] Navarro V, Shi L. The political context of social inequalities and health. *International Journal of Health Services*. 2001 1;31(1):1–21. 10.2190/1GY8-V5QN-A1TA-A9KJ 11271636

[24] Khubchandani J, Soni A, Fahey N, Raithatha N, Prabhakaran A, Byatt N, et al. Caste matters: perceived discrimination among women in rural India. *Archives of women’s mental health*. 2018 4 1;21(2):163–70. 10.1007/s00737-017-0790-1 29034410 PMC5857209

[25] Mishra PS, Syamala T. Multiple Vulnerabilities in Utilising Maternal and Child Health Services in Uttar Pradesh, India. Econ Polit Wkly. 2020;55(43):45–52.

[26] Borooah V. Inequality in health outcomes in India: the role of caste and religion. In Thorat S.& Newman K.S.(Eds.), *Blocked by caste—Economic discrimination in Modern India* (pp. 179–207). (New Delhi: Oxford University Press, 2010). 2010:179–207. 10.1107/S0108270110036887 20921621

[27] Gwatkin DR, Bhuiya A, Victora CG. Making health systems more equitable. *The Lancet*. 2004 10 2;364(9441):1273–80. 10.1016/S0140-6736(04)17145-6 15464189

[28] Mohanty SK, Srivastava A. Out-of-pocket expenditure on institutional delivery in India. Health policy and planning. 2013 5 1;28(3):247–62. 10.1093/heapol/czs057 22709923

[29] Prinja S, Bahuguna P, Gupta R, Sharma A, Rana SK, Kumar R. Coverage and financial risk protection for institutional delivery: how universal is provision of maternal health care in India?. PloS one. 2015 9 8;10(9):e0137315. 10.1371/journal.pone.0137315 26348921 PMC4562661

[30] Mishra S, Mohanty SK. Out-of-pocket expenditure and distress financing on institutional delivery in India. International journal for equity in health. 2019 12;18(1):99. 10.1186/s12939-019-1001-7 31238928 PMC6593606

[31] Mohanty SK, Kastor A. Out-of-pocket expenditure and catastrophic health spending on maternal care in public and private health centres in India: a comparative study of pre and post national health mission period. Health Economics Review. 2017 12 1;7(1):31. 10.1186/s13561-017-0167-1 28921477 PMC5603466

[32] Hunter BM, Bisht R, Chakravarthi I, Murray SF. Demand-side financing and promotion of maternal health: what has India learnt?. *Economic and Political Weekly*. 2014 1 11:66–73.

[33] Lagarde M, Haines A, Palmer N. Conditional cash transfers for improving uptake of health interventions in low-and middle-income countries: a systematic review. *JAMA*. 2007 10 24;298(16):1900–10. 10.1001/jama.298.16.1900 17954541

[34] Lim SS, Dandona L, Hoisington JA, James SL, Hogan MC, Gakidou E. India’s Janani Suraksha Yojana, a conditional cash transfer programme to increase births in health facilities: an impact evaluation. *The Lancet*. 2010 6 5;375(9730):2009–23. 10.1016/S0140-6736(10)60744-1 20569841

[35] Purnell TS, Calhoun EA, Golden SH, Halladay JR, Krok-Schoen JL, Appelhans BM, et al. Achieving health equity: closing the gaps in health care disparities, interventions, and research. *Health Affairs*. 2016 8 1;35(8):1410–5. 10.1377/hlthaff.2016.0158 27503965

[36] Chaturvedi S, Randive B, Diwan V, De Costa A. Quality of obstetric referral services in India’s JSY cash transfer programme for institutional births: a study from Madhya Pradesh province. *PloS One*. 2014;9(5). 10.1371/journal.pone.0096773 24810416 PMC4014551

[37] India, U. N. F. P. A. Concurrent assessment of Janani Suraksha Yojana (JSY) in selected states *UNFPA* 2009.

[38] Carvalho N, Rokicki S. The impact of India’s Janani Suraksha Yojana conditional cash transfer programme: A replication study. *The Journal of Development Studies*. 2019 5 4;55(5):989–1006.

[39] Powell-Jackson T, Mazumdar S, Mills A. Financial incentives in health: New evidence from India’s Janani Suraksha Yojana. *Journal of health economics*. 2015 9 1;43:154–69. 10.1016/j.jhealeco.2015.07.001 26302940

[40] Randive B, San Sebastian M, De Costa A, Lindholm L. Inequalities in institutional delivery uptake and maternal mortality reduction in the context of cash incentive program, Janani Suraksha Yojana: results from nine states in India. *Social Science & Medicine*. 2014 12 1;123:1–6. 10.1016/j.socscimed.2014.10.042 25462599

[41] Vellakkal S, Reddy H, Gupta A, Chandran A, Fledderjohann J, Stuckler D. A qualitative study of factors impacting accessing of institutional delivery care in the context of India’s cash incentive program. *Social Science & Medicine*. 2017 4 1;178:55–65. 10.1016/j.socscimed.2017.01.059 28199860 PMC5360172

[42] Thorat S, Attewell P. The legacy of social exclusion: A correspondence study of job discrimination in India. *Economic and political weekly*. 2007 10 13:4141–5.

[43] Ito T. Caste discrimination and transaction costs in the labor market: Evidence from rural North India. Journal of development Economics. 2009 3 1;88(2):292–300.

[44] Prakash A. Dalit Capital-State, Markets and Civil Society in Urban India. Taylor & Francis Limited; 2014.

[45] Deshpande A. Does caste still define disparity? A look at inequality in Kerala, India. American Economic Review. 2000 5;90(2):322–5.

[46] Kijima Y. Caste and tribe inequality: evidence from India, 1983–1999. *Economic Development and Cultural Change*. 2006 1;54(2):369–404.

[47] Karthick V, and Madheswaran S. Access to Formal Credit in the Indian Agriculture: Does Caste matter? *Journal of Social Inclusion Studies*, 2018 4(2), 169–195. 10.1177/2394481118814064.

[48] Kulkarni P. M. & Baraik V. K. Utilisation of healthcare services by Scheduled Castes in India (*Working Paper IIDS)*. New Delhi: National Family Health Survey 2003.

[49] Nayar KR. Social exclusion, caste & health: a review based on the social determinants framework. *Indian Journal of Medical Research*. 2007 10 1;126(4):355. 18032810

[50] Sabharwal NS. Caste, religion and malnutrition linkages. *Economic and Political Weekly*. 2011 12 10:16–18.

[51] Ministry of Health and Family Welfare (MOHFW). National Rural Health Mission (2005–2012), Mission Document. 16468284

[52] Blinder AS. Wage discrimination: reduced form and structural estimates. *Journal of Human resources*. 1973 10 1:436–55.

[53] Oaxaca R. Male-female wage differentials in urban labor markets. *International economic review*. 1973 10 1:693–709.

[54] Fairlie RW. An extension of the Blinder-Oaxaca decomposition technique to logit and probit models. *Journal of economic and social measurement*. 2005 1 1;30(4):305–16.

[55] Bora J.K., Raushan R. & Lutz W. The persistent influence of caste on under-five mortality: Factors that explain the caste-based gap in high focus Indian states. *PLoS ONE*. 2019 14(8): e0211086. 10.1371/journal.pone.0211086 31430275 PMC6701792

[56] Saroha E, Altarac M, Sibley LM. Caste and maternal health care service use among rural Hindu women in Maitha, Uttar Pradesh, India. Journal of midwifery & women’s health. 2008 9 1;53(5):e41–7. 10.1016/j.jmwh.2008.05.002 18761290

[57] Vellakkal S, Gupta A, Khan Z, Stuckler D, Reeves A, Ebrahim S, et al. Has India’s national rural health mission reduced inequities in maternal health services? A pre-post repeated cross-sectional study. *Health Policy and Planning*. 2017 2 1;32(1):79–90. 10.1093/heapol/czw100 27515405 PMC5886191

