# Supplementary material for: Impact of socio-economic inequity in access to maternal health benefits in India: Evidence from Janani Suraksha Yojana using NFHS data
Source: PLoS One. 2021 Mar 11;16(3):e0247935. doi: 10.1371/journal.pone.0247935 (PMC7951864; doi:10.1371/journal.pone.0247935)
Supplement: S1 File — (DOCX) [file pone.0247935.s001.docx]

**Descriptive statistics used for the Fairlie decomposition method**

| **Sl. No.** | **Variable name** | **Variable description** | **SC/ST** | | **Non-SC/ST** | |
| --- | --- | --- | --- | --- | --- | --- |
|  |  |  | **Mean** | **Std. Dev.** | **Mean** | **Std. Dev.** |
|  | **Dependent variable** | | | | | |
| 1. | Access to JSY | If women have access to JSY then =1, otherwise=0 | 0.4887 | 0.4999 | 0.3986 | 0.4896 |
|  | **Independent variables** | | | | | |
| 2. | **Type of birth** |  |  |  |  |  |
|  | Caesarean birth | if women's childbirth by Caesarean birth=1, normal birth=0 | 0.0960 | 0.2945 | 0.1706 | 0.3762 |
| 3. | **Birth order of the child** | | | | | |
|  | 2 and more | If woman’s child birth order is 2 and above=1, 1^st^ child=0 | 0.6558 | 0.4751 | 0.6119 | 0.4873 |
| 4. | **Age-group of the woman** | | | | | |
|  | Age 15-17 | If woman’s age is between 15-17=1, otherwise=0 | 0.0034 | 0.0580 | 0.0026 | 0.0508 |
|  | Age 18-30 | If woman’s age is between 18-30=1, otherwise=0 | 0.4278 | 0.4948 | 0.4395 | 0.4963 |
|  | Age above 30 | If woman’s age is above 30=1, otherwise=0 | 0.5688 | 0.4952 | 0.5579 | 0.4966 |
| 5. | **Education of the woman** | | | | | |
|  | Illiterate | if woman is illiterate=1, otherwise=0 | 0.3586 | 0.4796 | 0.2811 | 0.4496 |
|  | Primary | if woman is educated up to 5^th^ standard=1, otherwise=0 | 0.1689 | 0.3747 | 0.1308 | 0.3372 |
|  | Secondary | if woman is educated up to 10^th^ standard=1, otherwise=0 | 0.4184 | 0.4933 | 0.4702 | 0.4991 |
|  | Higher secondary and above | if woman is educated up to 12^th^ standard and above=1, otherwise=0 | 0.0541 | 0.2263 | 0.1178 | 0.3224 |
| 6. | **Occupation of the woman** | | | | | |
|  | Unemployed | if woman is unemployed= 1, otherwise=0 | 0.1165 | 0.3209 | 0.1413 | 0.3484 |
|  | Organised | if woman employed in organised sector=1, otherwise=0 | 0.0060 | 0.0771 | 0.0049 | 0.0699 |
|  | Unorganised | if woman employed in unorganised sector=1, otherwise=0 | 0.0464 | 0.2103 | 0.0273 | 0.1630 |
| 7. | **Occupation of the Husband** | | | | | |
|  | Unemployed | if husband is unemployed=1, otherwise=0 | 0.0070 | 0.0833 | 0.0071 | 0.0838 |
|  | Organised | if husband is employed in organised sector=1, otherwise=0 | 0.0223 | 0.1476 | 0.0423 | 0.2013 |
|  | Agriculture | if husband is employed in agriculture sector=1, otherwise=0 | 0.0700 | 0.2552 | 0.0477 | 0.2130 |
|  | Unorganised | if husband is employed in unorganised sector=1, otherwise=0 | 0.0693 | 0.2540 | 0.0764 | 0.2657 |
| 8. | **Wealth quintile** | | | | | |
|  | Poor | if women belong to poorer and poorest wealth quintiles =1, otherwise=0 | 0.6230 | 0.4846 | 0.4217 | 0.4938 |
|  | Middle | if women belong to middle wealth quintile=1, otherwise=0 | 0.1874 | 0.3902 | 0.2070 | 0.4051 |
|  | Rich | if women belong to richest and richer wealth quintiles =1, otherwise=0 | 0.1896 | 0.3920 | 0.3713 | 0.4832 |
| 9. | **Religion** | | | | | |
|  | Hindu | If woman belongs to Hindu=1, otherwise=0 | 0.7109 | 0.4533 | 0.7307 | 0.4436 |
| 10. | **Place of residence** | | | | | |
|  | Rural | if woman resides in rural=1, otherwise=0 | 0.8260 | 0.3791 | 0.7241 | 0.4470 |
| 11. | **Media** | | | | | |
|  | Radio | if woman got expose to radio=1, otherwise=0 | 0.1342 | 0.3408 | 0.1498 | 0.3569 |
|  | TV | if woman got expose to TV=1, otherwise=0 | 0.5480 | 0.4977 | 0.6234 | 0.4845 |
|  | Newspaper | if woman got expose to news=1, otherwise=0 | 0.1313 | 0.3377 | 0.1988 | 0.3991 |
