# Supplementary material for: Impact of socio-economic inequity in access to maternal health benefits in India: Evidence from Janani Suraksha Yojana using NFHS data
Source: PLoS One. 2021 Mar 11;16(3):e0247935. doi: 10.1371/journal.pone.0247935 (PMC7951864; doi:10.1371/journal.pone.0247935)
Supplement: S2 File — (DOCX) [file pone.0247935.s002.docx]

**Table 2. Percentage distribution of childbirth by place of delivery in major states of India and the differences between 2005-06 and 2015-16**

| **Indian States** | **Home** | | | **Public sector** | | | **Private sector** | | |
| --- | --- | --- | --- | --- | --- | --- | --- | --- | --- |
|  | **2015** | **2005** | **Diff** | **2015** | **2005** | **Diff** | **2015** | **2005** | **Diff** |
| Andhra Pradesh | 8.4 | 35.3 | -27.0 | 38.3 | 24.1 | 14.2 | 53.3 | 40.5 | 12.8 |
| Arunachal Pradesh | 47.5 | 71.0 | -23.5 | 42.9 | 19.8 | 23.1 | 9.6 | 9.2 | 0.5 |
| Assam | 29.3 | 77.6 | -48.3 | 60.1 | 13.0 | 47.1 | 10.7 | 9.5 | 1.2 |
| Bihar | 36.0 | 80.1 | -44.0 | 47.8 | 3.5 | 44.3 | 16.2 | 16.5 | -0.3 |
| Chhattisgarh | 29.7 | 85.7 | -56.0 | 55.9 | 6.7 | 49.2 | 14.4 | 7.4 | 6.9 |
| Gujarat | 47.1 | 11.1 | 36.0 | 14.0 | 32.7 | -18.7 | 38.9 | 56.2 | -17.3 |
| Haryana | 19.5 | 64.3 | -44.8 | 52.0 | 13.9 | 38.2 | 28.5 | 21.8 | 6.6 |
| Himachal Pradesh | 23.2 | 56.8 | -33.6 | 61.9 | 37.2 | 24.8 | 14.9 | 6.0 | 8.9 |
| Jammu & Kashmir | 13.8 | 49.6 | -35.8 | 78.6 | 41.2 | 37.4 | 7.6 | 9.2 | -1.6 |
| Jharkhand | 37.9 | 81.6 | -43.7 | 41.9 | 3.4 | 38.6 | 20.2 | 15.1 | 5.1 |
| Karnataka | 5.6 | 36.0 | -30.4 | 61.5 | 35.0 | 26.5 | 32.9 | 30.1 | 2.9 |
| Kerala | 0.1 | 0.6 | -0.5 | 38.4 | 35.6 | 2.7 | 61.5 | 63.8 | -2.2 |
| Madhya Pradesh | 19.0 | 73.7 | -54.7 | 69.6 | 18.4 | 51.2 | 11.4 | 7.9 | 3.5 |
| Maharashtra | 9.6 | 35.2 | -25.6 | 49.0 | 26.6 | 22.4 | 41.4 | 38.2 | 3.2 |
| Manipur | 30.7 | 54.1 | -23.5 | 45.8 | 36.1 | 9.7 | 23.5 | 9.8 | 13.7 |
| Meghalaya | 48.4 | 71.0 | -22.6 | 39.7 | 19.7 | 20.0 | 12.0 | 9.3 | 2.7 |
| Mizoram | 19.9 | 40.1 | -20.2 | 64.0 | 51.7 | 12.3 | 16.1 | 8.2 | 7.9 |
| Nagaland | 67.1 | 88.4 | -21.2 | 25.2 | 7.3 | 17.8 | 7.7 | 4.3 | 3.4 |
| Orissa | 14.2 | 64.2 | -50.0 | 76.2 | 28.9 | 47.3 | 9.6 | 6.9 | 2.7 |
| Punjab | 9.5 | 48.6 | -39.1 | 51.7 | 12.3 | 39.4 | 38.9 | 39.1 | -0.2 |
| Rajasthan | 15.9 | 70.4 | -54.5 | 63.6 | 19.0 | 44.6 | 20.5 | 10.6 | 9.9 |
| Sikkim | 5.3 | 52.7 | -47.4 | 82.7 | 44.6 | 38.1 | 12.0 | 2.7 | 9.3 |
| Tamil Nadu | 1.0 | 12.1 | -11.1 | 66.7 | 48.2 | 18.5 | 32.3 | 39.7 | -7.4 |
| Tripura | 20.0 | 52.9 | -32.9 | 69.2 | 43.2 | 25.9 | 10.8 | 3.9 | 7.0 |
| Uttar Pradesh | 31.9 | 79.4 | -47.5 | 44.7 | 6.6 | 38.1 | 23.4 | 14.0 | 9.4 |
| Uttarakhand | 31.2 | 67.2 | -36.0 | 43.9 | 15.8 | 28.1 | 24.9 | 17.0 | 8.0 |
| West Bengal | 24.4 | 57.8 | -33.5 | 56.9 | 31.9 | 25.1 | 18.7 | 10.3 | 8.4 |
| Telangana | 7.9 | (NA) | - | 30.8 | (NA) | - | 61.4 | (NA) | - |
| **India** | 20.9 | 61.2 | -40.4 | 52.2 | 18.1 | 34.2 | 26.9 | 20.7 | 6.2 |

Source: NFHS-3 & 4, 2005-6 and 2015-16.
