# Supplementary material for: Impact of socio-economic inequity in access to maternal health benefits in India: Evidence from Janani Suraksha Yojana using NFHS data
Source: PLoS One. 2021 Mar 11;16(3):e0247935. doi: 10.1371/journal.pone.0247935 (PMC7951864; doi:10.1371/journal.pone.0247935)
Supplement: S3 File — (DOCX) [file pone.0247935.s003.docx]

**Impact of socio-economic inequity in access to maternal health benefits in India: Evidence from *Janani Suraksha Yojana* using NFHS data**

**Abstract**

**Background:** Caste plays a significant role in Indian society and it influences women to health care access in the community. The implementation of the maternal health benefits scheme in India is biased due to caste identity. In this context, the paper investigates access to *Janani Suraksha Yojana* (JSY) among social groups to establish that caste still plays a pivotal role in Indian society. Also, this paper aims to quantify the discrimination against Scheduled Castes/Scheduled Tribes (SCs/STs) in accessing JSY.

**Methods:** This paper uses a national-level data set of both NFHS-3 (2005-06) and NFHS-4 (2015-16). Both descriptive statisticsand the Fairlie decomposition econometric modelhave been used to measure the explained and unexplained differences in access to JSY between SCs/STs and non-SCs/STs groups.

**Results:** Overall, the total coverage of JSY in India is still, 36.4%. It is found that 72%of access to JSY is explained by endowment variables. The remaining unexplained percentage (28%) indicates that there is caste discrimination (inequity associated with social-discrimination) against SCs/STs in access to JSY. The highest difference (54%) between SCs/STs and non-SCs/STs in access to JSY comes fromwealth quintile,with the positive sign indicating that the gap between the two social groups is widening.

**Conclusion and Discussion:** It is necessary for the government to implement a better way to counter the caste-based discrimination in access to maternal health benefits. In this regard, *ASHA* and *Anganwadi* workers must be trained to reduce the influence of dominant caste groups as well as they must be recruited from the same community to identify the right beneficiaries of JSY and in order to reduce inequity associated with social-discrimination.

**Keywords:** Maternal Healthcare, Caste discrimination, *Janani Suraksha Yojana*, India

**Introduction**

One of the Sustainable Development Goals (SDG) is to reduce the global maternal mortality ratio (MMR) to less than 70 per 100,000 live births by 2030. Worldwide, about 295,000 maternal deathswere registered during 2017, of which most (approximately 86%) were reported from Sub-Saharan Africa and Southern Asia (1). While Southern Asia accounts for nearly one-fifth of all maternal deaths, theregion hasregistered the greatest overall reduction in MMR by nearly 60%,that is, from 384 to 157 deaths during pregnancy and childbirth (1). However, the Indian side of the story is slightly different from that of the other Southern Asian countries. It is heartening that the MMR of India has declined from 167 in 2011-2013 to 130 in 2014-2016 and further, from 122 in 2015-17 to 113 in 2016-18. The MMR declinedby 6.2% in the country during the period 2014-2016 to 2015-2017, and 2015-17 to 2016-18 respectively, paving the way for the SDG target to be achieved much before the due year of 2030 (2). As per the bulletin, nearly 2,000 maternal deaths were averted per year during this period. This success story was made possible through the concerted efforts of the Indian government since the launch of the National Rural Health Mission (NRHM-2005) / National Health Mission (NHM-2013). Increased access to quality healthcare and wide coverage of health services under this scheme have majorly contributed to the declinein MMR. Due to this push factor, the share of institutional deliveries, including in private facilities,rose to 79% in 2016 from 18% in 2005 (3, 18).

Despite these achievements, the utilization of maternal healthcare services is still low in India (5).This is due to sluggishness in healthcare progress, uneven distributionof the healthcare services, and concentration of the services in one particular place, region and group for various socio-economic groups (8, 13). Women belonging to poor, marginalized, and disadvantaged groups face greater hardships in access to healthcare services than those from rich, non-marginalized, and advantaged groups (19, 9, 32, 21, 30, 24). For instance, far more (95%) of the economically well-off households receive institutional delivery servicesthan the worse-off households (59%). In terms of social groups, 68%, 78%, and 80%of the women belonging to Scheduled Tribes (STs), Scheduled Castes (SCs), and Other Backward Classes (OBCs) respectively receive institutional deliveryservices, whereas as many as 83% women belonging to Forward Castes (FCs)receive those services. The availability of skilled birth attendants during pregnancy among women varies in the same pattern (3-4). Therefore understanding these social and structural factors underlying MCH services required an assessment and also studying the distribution of healthcare benefits across socio-economic groups becomes imperative in the Indian context. A few studies have reported massive differences in access to healthcare benefits within and between socio-economic groups (16, 25). However, a dearth of literature was found in order to understand the inequity associated with socio-discrimination in maternal health benefits scheme in India. With this background, this paperexamines the disadvantage suffered in receiving maternal health benefits by the SC and ST women. The study also examines the determinants of access to maternal health benefits in India.

**Issues in access to maternal health benefits in India**

In general, access to maternal health is influenced by many factors. These can be broadly grouped as supply-side and demand-side factors. Demand-side factors that influence inequity in the use of and access to maternal healthcare services are recognized as the socio-economic and contextual factors (8, 51). Further, these factors are also underlying with women’s social determinists of health in society [6-9]. However, from the supply-side, inadequacy of the institutional structure and the underlying systems in providing services is a barrier (18). Along with that, there is an enormous lack of political willregarding remedying the social health inequities (32). The tremendous inequity in access to maternal health among socio-economic groups, as well as low-, middle- and high-income groups, is a major global concern (34, 29, 15). To address this issue, the WHO launched a ‘Commission on Social Determinants of Health’ in 2008 (15). This health policy framework aims to reduce health inequity among the most vulnerable and marginalized groups across the world. In India, Scheduled Castes and Scheduled Tribes women are the most deprived and marginalized groups in seeking healthcare services compared to their counterparts [8, 49, 52].

Seeking institutional delivery by poor, disadvantaged, and rural pregnant women face multiple burdens [21, 25, 52]. Financial burden is one of the fundamental factors which restrict pregnant women from delivering their babies at healthcare institutions (19, 12). Women who deliver childbirth at health institutions bear out-of-pocket expenditure which ranges from arranging a vehicle to go to the hospital, spend on medicine, staying at the health center, and many more [12, 20-21]. In India, still, after a huge investment in the public health system, the institutional delivery remains high across states, regions, and socio-economic groups [53-56]. Although achieving Universal Health Coverage (UHC) is one of the major SDGs that intends to provide financial risk protection to all eligible beneficiaries along with access to quality and essential healthcare services. Many low- and middle-income countries have introduced UHC programmes for the benefit of their citizens. This supports in reducing health inequity and promoting the use of healthcare access among the poor and marginalized communities across countries (27-28, 22, 37).India too introduced a conditional cash transfer scheme called *Janani Suraksha Yojana* (JSY) in2005 to promote institutional delivery and post-natal care to reduce MMR and child mortality rates (CMR). This is one of the most extensive demand-side financing programmes launched in India (22). It ensures safe delivery for all women aged 19 and above who belong to anSC/STgroup and are below the poverty line (BPL) at the time ofchild delivery. ASHA (Accredited Social Health Activists) health workers play a significant role in ensuring community health by tracking women from pregnancy to childbirth and post-natal care. Each ASHA is engaged with the JSY scheme as a link between the government’s health system and poor pregnant women from the community. Studies have shown that financial assistance during pregnancy has led to greater utilization of maternal services (23, 14, 36, 31). However, all eligible beneficiaries do not receive the cash payment under the JSY scheme, and low coverage and under-utilization of the scheme have been found to prevail across the socio-economic groups (41, 38). Several studies have also reported that the practice of laying of conditions for availing the JSY cash transfer scheme during pregnancy prevents many eligible women from accessing JSY (18, 28, 36, 41). Therefore it becomes imperative to understand the low-coverage of JSY across the eligible beneficiaries among SC/ST women and the factors behind it.

Asthe Indian society is caste-based, the caste factor plays a significantrole in all kinds of economic outcomes. Caste, as a predictor of economic outcomes, can also be correlated with occupation and employment (42, 44-45), income and expenditure (47), capital (46), and access to credit (43). As the healthcare system is considered as one among economic outcomes, it is expected that caste can play a major role in healthcare access. Studies such as those by Kulkarni and Baraik (26), Borooah et al. (13), and Acharya (6-7) have exposed that caste influences access to healthcare systems. Social discrimination in health and healthcare practices puts women from poor and disadvantaged groups ata high risk (8). Other studies such asthose by Nayar (33), Borooah (12), and Sabharwal (39) have also identified that lower caste women face discrimination in accessing essential reproductive healthcare services compared to higher caste women. To the best of our knowledge, no studyhas so far reported that caste discrimination prevails in the access to the JSY cash payment scheme among the social groups in India. Hence, this study aims to understand the disparities and discrimination prevalent against the poor, marginalized, and disadvantaged women in the Indian society as they seek to avail the JSY cash payment scheme. The analysis seeks empirical evidence on how health policymakers can enhance the programme and revisit the implementation strategy.

**Materials and Methods**

On the completion of ten years of the National Rural Health Mission (NRHM) (48), under which the JSY programme was launched to promote institutional delivery among the poor and marginalized members of the community, a need was felt to focus on the achievements of the JSY scheme and explore the scope for improvement. In order to study the access to JSYamong different social groups and states, weused unit-level data extracted from the National Family Health Survey (NFHS). The NFHS is a large-scale and multi-round survey conducted in a representative sample of households across India by the International Institute for Population Sciences (IIPS), Mumbai, since 1992-93. So far, four rounds have been conducted, the latest onehaving been surveyed during 2015-16 (NFHS-4). The NFHS provides vital data on health and family welfare and other issues related to them at both national and state levels. For the descriptive statistics, we used data from NFHS-3 (4) and NFHS-4 (3), where the sample sizes of women aged 15-49 years were 124,385, and 699,686 respectively. To analyze the determinants of access to JSY and to decompose the access to JSY across social groups, thestudy used only NFHS-4.The samples used werethe births that took place during five years preceding the survey, making the total sample size equal to 259,627. The unit-level data from the kids-file is used that covered 190898 births (last birth) in five years preceding the survey. Of these births, 148746 births were conducted in the health facilities of which JSY assistance was provided to the mothers of 63665 births. Fig 1 gives details about how the sample is drawn for the analysis. Further, the categorization of EAG (Empowered Action Group) states and Non-EAG states has followed the methodology of Sample Registration System, the office of registrar general of India, which publishes Special Bulletin on MMR [2] The Maternal Mortality Bulletin (2014-2016) categorizes the Indian states as Empowered Action Group plus (EAG plus), Southern states, and Others in order to better understand MMR and its distributional regional patterns. The EAG plus states are Bihar, Jharkhand, Madhya Pradesh, Chhattisgarh, Odisha, Rajasthan, Uttar Pradesh, Uttarakhand, and Assam, while the southern states are Andhra Pradesh, Telangana, Karnataka, Kerala, and Tamil Nadu. The remaining states/UTs are categorized as ‘others.

**Fig 1 Schematic presentation of children born in five years preceding the survey by place of delivery and JSY beneficiaries in India, NFHS-4, 2015-16**

**Ethics Statement**

The data used in the study is available in the public domain. The National Family Health Survey (NFHS) is the largest health survey in the world, which is conducted by the International Institute for Population Sciences (IIPS), Mumbai, under the Ministry of Health and Family Welfare, Government of India. The first survey was conducted in 1992. The 2^nd^ and the 3^rd^ roundswere conducted in 1998 and 2005-06 respectively. The 4^th^ and latest round was conducted during 2015-16.

**Econometric Methodology**

The decomposition method was first developed by Blinder (10) and Oaxaca (35) to study the discrimination between males and females in the labor market. Later, the application was extended to and applied in other sectorsto compare advantaged and disadvantaged groups. Most studies apply the linear model to decompose the outcome variable between advantaged and disadvantaged groups. Since access to JSY is usually not random but based on specific socio-economic characteristics, the dependent variable is binary (access to JSY = 1, otherwise = 0). Hitherto, as per our knowledge, there have been no studies that have decomposed JSY as a health outcome. Our study is the first one to decompose the access to JSY into explained and unexplained differencesbetween two groups using the non-linear Fairlie decomposition model (17). For the analysis, we combined both SCs and STs and referred to them as SC/ST and referred to the rest of the caste groups (both OBCs and FCs) as non-SC/ST. As mentioned in the Fairlie decomposition model (17), the gap in the average value of the dependent variables between non-SCs/STs and SCs/STs, $i.e.Y^{n}-Y^{s}$(i.e. access to JSY) can be written as:

$$Y^{n}-Y^{s}=\left[ \left( X^{n}-X^{s} \right)\hat{\beta}^{n} \right]+\left[ X^{s}\left( \hat{\beta}^{n}-\hat{\beta}^{s} \right) \right]$$

Unlike the Blinder-Oaxaca (10, 35) model, the Fairlie decomposition (17) follows the logistic distribution function because of the non-linear binary (1, 0) dependent variables. In the above equation, the first term in brackets denotes explained differences, that is, the gap between the two groups due to group differences in the distribution of the endowment variables.The second term represents unexplained differences that capture the gapdue to group differences in unmeasurable or unobserved endowments (see Bora et al., (11) for further understanding of Fairlie decomposition). Moreover, the descriptive analysis of the variables used in the study can be found in the supporting document (supporting file 1)

**Results and Discussion**

**Status of childbirth and place of delivery in India**

The Maternal Mortality Bulletin (2014-2016)categorizesthe Indian statesas Empowered Action Group plus (EAG plus), Southern states, and Others in order to better understand MMR. The EAG plus states are Bihar, Jharkhand, Madhya Pradesh, Chhattisgarh, Odisha, Rajasthan, Uttar Pradesh, Uttarakhand, and Assam, whilethe southern states are Andhra Pradesh, Telangana, Karnataka, Kerala, and Tamil Nadu. The remaining states/UTs are categorized as ‘others’. Since the commencement of NRHM and JSY in 2005, there has beenan increase in childbirth at homeas well as in public and private hospitals. JSY is mainly meant to encourage women to deliver in public hospitals. In this regard, the country has shown improvement in the last decade as the share of public hospital delivery increased from 18 to 52%while that of home deliverydeclined significantly from 61 to 21%from 2005 to 2015 (Table 1). Regarding childbirth at home, the southern states dida betterjobby bringing their share of home delivery down to 8%, which is lower than that of EAG plus states (29%) and otherstates (13%). This suggests that more healthcare facilities are available in the southern states.But whether they are affordable for everyone is a vital question that has to be answered. In the case of childbirth in public hospitals, all the states performedequally well, taking their shareto above 50%in 2015. The share of childbirth in private hospitals also increased at the national level by 6%between 2005 and 2015. This suggests that there is a need to revamp the policies to empower the public hospitals so as to reduce MMR due to home delivery as well as reduce exploitation by the private hospitals.

**Table 1. Distribution of childbirth by place of delivery in 2005 and 2015 (%)**

| **Sl.**  **No** | **State groups** | **Home Delivery** | | **Public Hospitals** | | **Private Hospitals** | |
| --- | --- | --- | --- | --- | --- | --- | --- |
|  |  | **2005** | **2015** | **2005** | **2015** | **2005** | **2015** |
| 1. | EAG states | 76.9 | 28.5 | 10.3 | 53.2 | 12.8 | 18.2 |
| 2. | Southern states | 25.8 | 7.6 | 34.3 | 50.4 | 39.9 | 42.0 |
| 3. | Other states | 48.4 | 13.2 | 24.8 | 50.9 | 26.7 | 35.9 |
|  | All India | 61.1 | 20.8 | 18.0 | 52.1 | 20.8 | 27.1 |

Source: Author’s calculation based on NFHS-3 (2005-06) and NFHS 4 (2015-16).

Major state-wise distribution of childbirth shows that the share of childbirth at home declined in almost every stateduring 2005-15 (Figure 1A). Kerala recorded the least number of childbirths at home, while Nagaland toppedthe list with over 60%of the childbirths taking place at home in 2015. Among the EAG plus states, Orissa recorded the least (15%) share of home delivery, whereas Jharkhand recorded the highest (38%) share in 2015. Compared with the other two groups, EAG plus states had more childbirths at home. In the case of public hospital delivery, except the EAG states of Jharkhand, Uttar Pradesh, and Uttarakhand, the other EAG states exhibited better results with more than 50%public hospital deliveriesin 2015 (Figure 1B). Among the southern states, Tamil Nadu recorded the most (67%) number of childbirthsin public hospitals, followed by Karnataka (64%). Among all the states, Sikkim recordedthe highest(85%)number of childbirthsin public hospitals and Nagaland the lowest (7%). In a few states, both childbirth at home and at public hospitals show contrary results. For instance, Nagaland recorded the least number of births in hospitals whether private or public, whereas Kerala recorded the most number of private hospital deliveries. These results strongly suggest that these states have to improve public healthfacilities.

Women from Kerala, Andhra Pradesh, and Gujarat prefer private hospitals for child delivery (Figure 1C). Though the number of childbirthsinpublic hospitals has increased in the last decade, the share of private hospitals is also very high in some states. Overall, improvement has been undoubtedly witnessed in institutional delivery whether inpublic or private hospitals, and it has been possible only through the implementation of the demand-side financing programme to empower all pregnant women.

**Figure 1. State-wise distribution of childbirth by place of delivery in India**

However, it is necessary to understand whether all women, irrespective of caste, religion, and other related circumstances, can access JSY. From Figure 2, it is clearly visible that there were widespread persistent disparities in childbirth at homeand at public and private institutions among social groups between 2005 and 2015. During this period, deliveries at home reduced drastically. The rate of public delivery increased manifoldacross the groups, and the rate of private hospital delivery also increased to some extent among various social groups. Yet, differences in access to JSY exist among social groups. Home delivery among SCs/STsis higher than among non-SCs/STs, whereas private hospital delivery is lower. Childbirth in public hospitalsis high among SCs/STs and that has given them access to JSY financial assistance. However, a few studies are sceptical and say that these differences in access to JSYare,in fact, due to caste practices in India (28, 41).

**Figure 2: Distribution of institutional delivery (%) across social groups in India**

**Access to JSY among social groups**

Women in India who are poor, marginalized, and live in remote areasface financial hardships in access to healthcare institutions, which in turn leads to high levels of MMR. In order to address this, JSY, a cash transfer programme aimed at reducing MMR and facilitating childbirth at public hospitals, was introduced in 2005. A decadeafter its inception, its national coverage stood at 36.4%in 2015 (Table 2). The eligibility criteria for pregnant women to avail JSY vary across low and high performing states in India. In the low-performance states, all pregnant women who undergo childbirth at the public or accredited private institutions are eligible to receive assistance under the scheme, whereas in the high-performance states, all pregnant women who belong to BPL households, belong to SC/ST, are 19 years or aboveof age, and undergo childbirth at public or accredited private health institutions up to second births, are eligible.

**Table 2. Share of access to JSY among social groups by major states during 2015-16(%)**

| **Sl. No.** | **States** | **SC/ST** | **Non-SC/ST** | **Total** |
| --- | --- | --- | --- | --- |
| ***I*** | ***EAG Plus Assam states*** | ***36.8*** | ***62.6*** | ***57.3*** |
| 1. | Uttar Pradesh | 30.1 | 69.8 | 48.7 |
| 2. | Bihar | 26.4 | 73.4 | 53.9 |
| 3. | Uttarakhand | 28.9 | 69.6 | 49.4 |
| 4. | Chhattisgarh | 47.6 | 52.3 | 66.2 |
| 5. | Jharkhand | 39.9 | 59.9 | 41.6 |
| 6. | Madhya Pradesh | 38.5 | 61.1 | 61.1 |
| 7. | Rajasthan | 37.5 | 61.5 | 56.1 |
| 8. | Orissa | 48.3 | 51.1 | 72.6 |
| 9. | Assam | 34.0 | 65.1 | 66.1 |
| ***II*** | ***Southern states*** | ***35.6*** | ***63.7*** | ***19.8*** |
| 10. | Andhra Pradesh | 39.8 | 59.9 | 17.4 |
| 11. | Telangana | 35.7 | 62.7 | 11.9 |
| 12. | Karnataka | 42.7 | 56.1 | 19.9 |
| 13. | Tamil Nadu | 37.9 | 62.1 | 29.5 |
| 14. | Kerala | 22.1 | 77.6 | 20.4 |
| ***III*** | ***Other states*** | ***62.5*** | ***36.7*** | ***25.7*** |
| 15. | Arunachal Pradesh | 76.0 | 23.4 | 20.5 |
| 16. | Gujarat | 47.7 | 49.7 | 8.9 |
| 17. | Haryana | 54.1 | 45.7 | 13.5 |
| 18. | Himachal Pradesh | 50.9 | 48.8 | 13.1 |
| 19. | Jammu and Kashmir | 39.6 | 60.1 | 54.0 |
| 20. | Maharashtra | 53.9 | 45.8 | 8.7 |
| 21. | Manipur | 31.2 | 67.0 | 26.2 |
| 22. | Meghalaya | 99.6 | 0.4 | 28.0 |
| 23. | Mizoram | 99.1 | 0.3 | 47.5 |
| 24. | Nagaland | 98.7 | 1.3 | 29.4 |
| 25. | Punjab | 58.3 | 41.7 | 19.1 |
| 26. | Sikkim | 44.7 | 54.9 | 29.4 |
| 27. | Tripura | 67.9 | 31.7 | 32.6 |
| 28. | West Bengal | 53.2 | 43.6 | 28.7 |
|  | ***All India*** | ***37.6*** | ***61.8*** | ***36.4*** |

*Source: Author’s calculation based on NFHS-4 (2015-16).*

The national average of access to JSY is 36%, and only a few states such as all EAG plus states (above 41%), Mizoram (48%), and Jammu & Kashmir (54%) have crossed this average (Table 2). With 72.6%access to JSY, Orissa stands at the top, while with 8.7 and 8.9%access respectively,Maharashtra and Gujarat are the bottom. Since India faces an immense health inequity due to persisting socio-economic inequality, social discrimination in access to JSY is also associated with low accessto and under-utilization of healthcare services across states. At the social group level,the national share of SCs/STs in access to JSY is lower (38%) than that of non-SCs/STs (62%). Naturally, there is a variation at the state level in the access to JSY between SCs/STs and non-SCs/STs. Non-SCs/STs have a greater share than their SC/ST counterparts in both the EAG plus and the southern states in the total access to JSY. Arunachal Pradesh, Haryana, Himachal Pradesh, Maharashtra, Meghalaya, Mizoram, Nagaland, Punjab, Tripura, and West Bengal are the major states in whichthe access of SCs/STs to JSY is higher.SCs/STs in the rest of the states have a lower access to JSY than non-SC/STs. In many states, thereis a contradictionbetween the number of childbirths inpublic hospitalsand the access of SCs/STs to JSY. In other words, states childbirth in public hospitals among SCs/STs is higher show less accessof these social groups to JSY.This contradiction clearly shows that discrimination prevails in the access to JSY against SCs/STs.

**Determinants of Access to JSY by Caste**

From the above descriptive results, it is evident that access to JSY depends on caste. To further show that caste influences access to JSY, we applied the Fairlie decomposition method,followed by the Blinder-Oaxaca (10, 35) model. First, the model estimated the augmented logistic regression function separately for SCs/STs and non-SCs/STs. The dependent variables were dichotomous (1= access to JSY, 0= otherwise), while the group of independent variables were qualitative viz., delivery type, birth order type, level of woman’s education, employment and age, level of husband’s education and employment, quintile of household wealth, religion, and residential place (rural or urban). The rationale behind selectingthese independent variables is that JSY is an economic welfare scheme to help pregnant women during childbirth and may be affected by socio, economic, demographic, and geographic variables. Since this scheme promotes institutional delivery, it is meant for childbirth in public hospitals irrespective of whether it is a caesarean birth ora normal delivery. But the negative coefficients of both SC/ST and non-SC/ST women show that as compared to women having a normal delivery,those having a caesarean birth delivery have less likelihood of access to JSY (Table 3). The positive coefficients of the birth order, irrespective of caste, show that the delivery of the second and each successive child has more likelihood of access to JSY.

**Table 3. Determinants of access to JSY: *Logistic regression results***

| **Sl. No.** | **Variables**  **name** | **SC/ST** | | **Non-SC/ST** | |
| --- | --- | --- | --- | --- | --- |
|  |  | **Coefficients** | **Standard Error** | **Coefficients** | **Standard Error** |
| 1. | **Type of birth** *(Ref: normal birth)* | | | | |
|  | Caesarean birth | -0.852*** | (0.028) | -1.066*** | (0.021) |
| 2. | **Birth order of the child** *(Ref: 1^st^ child)* | | | | |
|  | 2 and more | 0.0009 | (0.021) | 0.062*** | (0.017) |
| 3. | **Age-group of the women** *(Ref: above 30)* | | | | |
|  | Age 15-17 | -0.793*** | (0.143) | -0.815*** | (0.138) |
|  | Age 18-30 | -0.146*** | (0.020) | -0.029** | (0.016) |
| 4. | **Education of the women***(Ref: illiterate)* | | | | |
|  | Primary | -0.029 | (0.029) | 0.014 | (0.026) |
|  | Secondary | -0.025 | (0.024) | -0.109*** | (0.021) |
|  | Higher secondary and above | -0.022*** | (0.044) | -0.453*** | (0.031) |
| 5. | **Occupation of the women***(Ref: organized sector)* | | | | |
|  | Unemployed | -0.102* | (0.057) | -0.051* | (0.037) |
|  | Unorganised | 0.019 | (0.071) | -0.061 | (0.057) |
| 6. | **Occupation of the Husband** *(Ref: organized sector)* | | | | |
|  | Unemployed | -0.269** | (0.114) | -0.088 | (0.091) |
|  | Agriculture | 0.059 | (0.067) | -0.055 | (0.050) |
|  | Unorganised | 0.169*** | (0.064) | 0.068 | (0.044) |
| 7. | **Wealth quintile** *(Ref: rich)* | | | | |
|  | Poor | 0.729*** | (0.029) | 0.867*** | (0.022) |
|  | Middle | 0.333*** | (0.028) | 0.507*** | (0.020) |
| 8. | **Religion** *(Ref: others)* | | | | |
|  | Hindu | 0.448*** | (0.022) | 0.279*** | (0.019) |
| 9. | **Place of residence** *(Ref: urban)* | | | | |
|  | Rural | 0.174*** | (0.025) | 0.303*** | (0.018) |
| 10. | **Media** | | | | |
|  | Radio | 0.039 | (0.026) | 0.049*** | (0.021) |
|  | TV | -0.049** | (0.022) | -0.066*** | (0.019) |
|  | Newspaper | -0.073*** | (0.028) | -0.209*** | (0.020) |
|  | Constant | -0.718*** | (0.044) | -0.904*** | (0.034) |
|  | Log likelihood | - 33775.728 | | -53559.386 | |
|  | Samples (N) | 51,896 | | 89,195 | |

Source: Author’s calculation based on NFHS-4 (2015-16).

Note: The dependent variable is access to JSY.

***p < 0.01, **p < 0.05, *p < 0.1.

Women belonging to the age group above 30 years have more likelihood of access to JSY than women less than 30 years of age. Onesignificant observationis that women in the age group of 15-17years have very less likelihood of access to JSY.Sincemarriageinthis age group is legally considered as child marriage, pregnant women in this age group likely do not seek the benefit of JSY. The coefficients of the educational level of women are negative for both SCs/STs and non-SCs/STs, which shows that education does not influence access to JSY. Despite the negatively significant coefficient of higher education in case of both SCs/STs and non-SCs/STs, the likelihood of access to JSY among SCs/STs increases only when they have higher secondary and abovelevels of education. This result highlights that either non-SC/ST pregnant women do not seek JSY as most of them prefer private hospitals over public hospitals orthat they do not need government hospital facilities. Compared to women working in the organized sector, the likelihood of access to JSY among unemployed women is less among both SCs/STs and non-SCs/STs. This underscores the need for the JSY policy to be revamped for the benefit of unemployed women too. The coefficients of occupation of the husband are seen to increase positively, irrespective of significance, as we go from the unemployed sector to the unorganized sector. This shows that husbands from both SCs/STs and non-SCs/STs are well aware of JSY due to their peer group workers.

When it comes to economic factors, wealth quintileis the most important factor determining access to JSY. The result shows that women from middle and poor quintiles have higher chances of accessing JSY than those from the richquintile. This is possibly due to poor women’spreferencefor government hospitals due to the belief that they are easily available, affordable, and good. In the case of religion, as the majority of the women are Hindus, they have more access to JSY than the non-Hindus.This indicates that women from the minority religions are deprived of access to JSY. Rural women are more likelyto access JSY than urban women irrespective of caste. However, the higher (0.303) likelihood ratio of rural non-SC/ST women shows that they have more access to JSY than their SC/ST counterparts (0.174). Access to JSY is possibly influenced by media contact as most welfare schemes arepublicized in the media for the benefit of the public. In the case of radio, the coefficients are positive and significant for non-SCs/STsbut not for SCs/STs. Both TV and newspapers have failed to influence households in access to JSY. This result strongly suggests the need forwelfare schemes to be advertised through TV and newspapers as they are the most important sources of information. From the above logistic regression results, it is clear that access to JSY depends on many socio-economic and other variables. Like with descriptive statistics, the regression results also show that there are differences in access to JSY between SCs/STs and non-SCs/STs.

**Decomposition of Access to JSY by Caste**

The Fairlie decomposition results are presented in Table 4.The results indicate that even after considering important variables,only up to 72% access to JSYcan be explained.This implies that there are still some other factors that influence access to JSY. As both Blinder-Oaxaca (10, 35) and Fairlie (17) elucidate, the remaining unexplained percentage (23%) is considered as discrimination coefficients due to the group differences between non-SC/STs and SC/STs.

**Table 4: Differences in JSY access between SC/ST and non-SC/ST**

| **Sl.**  **No.** | **Covariates** | **Access to JSY** | |
| --- | --- | --- | --- |
|  |  | **Coefficient** | **Percentages to the total (%)** |
| **Covariates contributions** | | | |
| 1. | Caesarian birth | -0.015*** | 22.2 |
| 2. | Birth order of the child | -0.0002*** | 0.4 |
| 3. | Age-group of the women | -0.0001** | 0.07 |
| 4. | Education of the women | -0.009*** | 12.5 |
| 5. | Occupation of the women | -0.001 | 2.0 |
| 6. | Occupation of the Husband | 0.002 | -2.2 |
| 7. | Wealth quintile | -0.038*** | 54.1 |
| 8. | Religion | 0.003*** | -4.5 |
| 9. | Place of residence | -0.007*** | 10.0 |
| 10. | Media | -0.004*** | 5.4 |
|  | **Total (=1 to 10)** | **-0.069** | **100** |
| **Decomposition results** | | | |
| 1. | Total explained gap | -0.069 | 71.9 |
| 2. | Total unexplained gap | -0.027 | 28.1 |
|  | **Total raw differentials (=1+2)** | **-0.096** | **100** |
| Mean prediction of Non-SC/ST | | 0.392 | |
| Mean prediction of SC/ST | | 0.489 | |
| Samples (N) | | 1,41,091 | |

Source: Author’s calculation based on NFHS-4 (2015-16).

Note: ***p < 0.01, **p < 0.05, *p < 0.1.

The contribution effect of each endowment showsthat the highest difference in access to JSY between SCs/STs and non-SCs/STs is explained by the wealth quintile.The positive sign indicates that the gap between these two groups is to the extent of 54%(Table 4). The next disadvantage to SC/ST households comes from caesarean birth, where there is a 22% gap between the two groups in access to JSY. This indicates that women from non-SC/ST households have more advantages than their SC/ST counterparts for child delivery. The educational level of women is also important in access to JSY as there is a 12.5% gap between the two groups. Even though most factors make a meagre contribution individually, the fact that the variables are positive shows that access to JSY is significantly inclined towards non-SC/STs. Thus, it is very evident that both SC and ST women face discrimination in access to JSY due to the evil caste hierarchy in India just like in other economic outcomes such as access to a job, access to credit, and access to health services.

**Conclusion and policy implications**

JSY is a cash transfer programme that aims to reduce MMR and CMR during childbirth by promoting institutional delivery and post-natal care. This paper shows that access to JSY varies across states and social groups and that there is a huge gap in accessto the scheme even among the beneficiary groups. The all-India coverage of JSY is 36.5%.Except in a few states like Orissa, Chhattisgarh, Assam, and Madhya Pradesh, where access to JSY is above 60%, access in the remaining states like Maharashtra, Gujarat, and Telangana is less than 36%. The difference in availing JSY services between SC/ST (37.6%) and Non-SC/ST (61.8%) women are 24.2 points. Even though a few states have a robust institutional delivery system,they do poorly in terms of access to JSY. While the scheme is expected to increase equity in the utilization of maternal healthcare services such as prenatal, natal, and post-natal care, the results reveal that there are inequalities in the distribution of JSY across social groups.

Though the programme was initially to launch in the low performing states like EAGs plus Assam where the MCH service coverage was poor, later, it extended to all states and union territories. Due to the JSY scheme, the MCH service utilization among marginalized women has increased [36, 38]. However, the difference between and within the social group has remained the same, and the difference found between the social groups like SC/ST and Non-SC/ST is huge.The paper finds that access to JSY is influenced by individual, household, and community-level factors. To access and utilize social welfare schemes in India, caste and class status are necessary rather than the necessity of services provided by either public or private hospitals. Social, economic, and demographic factors determine the access to JSY among social groups in the country. Most of the factors such as birth order of the children, age and education of the women, occupation of the husband, household’s wealth status, place of residence, and media for spreading awareness play a role in the under-utilization of and lowaccess to the programme. The Fairlie decomposition analysis (17) shows that a wide gap of around 54%exists between SC/ST and non-SC/ST households due to differences in wealth. It shows that access to JSY is highly influenced by economic status. Both descriptive and econometric analyses conclude that caste plays a major role in access to JSY in India. Due to thecaste hierarchy and the resultantdiscrimination, SC/ST women experience differential treatment at childbirth, leading to less economic and social well-being in the country (57). Further, the mother’s education has also contributed to a 12.5% gap between SC/ST and Non-SC/ST women in influencing to access the JSY services.

Certainly, programmes and policy interventions related to JSY have made an impact on service accessibility (23), but the social identity plays a role in maintaining the inequality of access and utilization of the services (18). However, earlier studies have clearly revealed that there exist huge differential and discrimination across social groups in accessing healthcare services in the community [49]. Here, this study also provides some plausible explanations for the above findings with quantifiable evidence. Our findings revealed that there is inequity associated with social-discrimination in receiving JSY services across social groups that could be seen with the bivariate and multivariate analysis. The JSY programme was aimed to reduce the inequity and inequality in MCH care services across class, caste, rural-urban, and state-regions; however, the health system level barriers and the social determinants of health that still, constraints in reducing the gap which prevails in accessing social welfare scheme [18, 28, 50].

Furthermore, our findings also revealed that demand-side financing needs a proper implementation strategy that is apparently perceived in caste-wise institutional delivery in India. Thus, strengthening the health system is required in order to improve the supply-side mechanisms and it is also needed to enhance the interpersonal communications with community health workers and stakeholders that is lacking in the public health system in India [50]. This may have the ultimate effect on covering the existing programme to everyone.

To rectify all those issues, and to maintain the balance between accessto and utilization of the scheme, our study has policy suggestions. The first is that since ASHA workers play a vital role in dealing with the community level issues from tracking pregnancy to childbirth, they have to be trained well to identify and avoid caste influences in access to JSY. Furthermore, both ASHA and Anganwadi workers must be encouraged to work together to identify the right beneficiariesof JSY. Finally, there needs to be a system of checks and balances in place to ensure that such problemsdo not arise in the first place. Only whenmeasures like these are put in place canprogrammes like JSY succeed in providing services to all women without any biases and discrepancies.

**Declarations**

**Ethics approval and consent to participate:** The data used in the study is available in the public domain with no identifiable information on the survey participants; therefore, no ethics statement is required for this work. The National Family Health Survey (NFHS) is the largest health survey in the world, which is conducted by the International Institute for Population Sciences (IIPS), Mumbai, under the Ministry of Health and Family Welfare, Government of India.

**Consent for publication:** Not applicable

**Availability of data and materials:** The study utilises secondary source of data which is freely available in public domain through <http://iipsindia.org>.

**Competing Interests:** The authors declare that they have no competing interests.

**Funding:** Authors did not receive any funding to carry out this research.

**Author’s Contribution:** The concept was drafted by PSM. PSM, KV and PKC contributed to the analysis design; PSM, PKC and KV advised on the paper and assisted in paper conceptualization; PSM, PKC, and K V contributed to the comprehensive writing of the article. All authors read and approved the final manuscript.

**Acknowledgments:** Not applicable

**Supporting Information files**

**Supporting file 1**

Descriptive statistics of main variables used in the OLS equation of access to JSY

**Supporting file 2**

Percentage distribution of childbirth by place of delivery in major states of India and the differences between 2005-06 and 2015-16

**References**

1. WHO. (2019, September 19). Retrieved from World Health Organaization: https://www.who.int/news-room/fact-sheets/detail/maternal-mortality. 2019 Sept.
2. *Special Bulletin on Maternal Mortality in India 2015-17.* New Delhi: *Sample Registration System, Office of Registrar General*, Vital Statistics Division, India 2019.
3. IIPS, ICF. National Family Health Survey (NFHS-4), 2015–16: India. Mumbai: International Institute for Population Sciences 2017.
4. IIPS, ORC-Macro. National Family Health Survey (NFHS-3), 2005–2006: India. Mumbai: International Institute for Population Sciences 2007.
5. Paul S, Paul S, James KS. Universalisation versus targeting in maternal and child health care provisioning: Evidence from India. *SSM-population health*. 2019 Dec 1;9:100502.
6. Acharya S. Caste and patterns of discrimination in rural public health care services. Blocked by Caste: Economic Discrimination in Modern India (New Delhi: Oxford University Press, 2010). 2010:208-29.
7. Acharya SS. Conceptualizing social discrimination in access to health services towards measurement-illustrating evidences from Villages in Western India. *Indian Emergency Journal*. 2011;6(2):14-27.
8. Baru R, Acharya A, Acharya S, Kumar AS, Nagaraj K. Inequities in access to health services in India: caste, class and region. *Economic and Political Weekly*. 2010 Sep 18:49-58.
9. Blas E, Gilson L, Kelly MP, Labonté R, Lapitan J, Muntaner C, Östlin P, Popay J, Sadana R, Sen G, Schrecker T. Addressing social determinants of health inequities: what can the state and civil society do?. *The Lancet*. 2008 Nov 8;372(9650):1684-9.
10. Blinder AS. Wage discrimination: reduced form and structural estimates. *Journal of Human resources.* 1973 Oct 1:436-55.
11. Bora J.K., Raushan, R. & Lutz, W. The persistent influence of caste on under-five mortality: Factors that explain the caste-based gap in high focus Indian states. *PLoS ONE*. 2019 14(8): e0211086. https://doi.org/10.1371/journal. pone.0211086
12. Borooah V. Inequality in health outcomes in India: the role of caste and religion. In S. Thorat& K.S. Newman (Eds.), *Blocked by caste—Economic discrimination in Modern India* (pp. 179–207). (New Delhi: Oxford University Press, 2010). 2010:179-207.
13. Borooah VK, Sabharwal NS, Thorat S. Gender and caste-based inequality in health outcomes in India. Indian Institute of Dalit Studies; New-Delhi (*Working Paper Series 7(3)*) 2012.
14. Chaturvedi S, Randive B, Diwan V, De Costa A. Quality of obstetric referral services in India's JSY cash transfer programme for institutional births: a study from Madhya Pradesh province. *PloS One*. 2014;9(5).
15. WHO Commission on Social Determinants of Health, World Health Organization. Closing the gap in a generation: Health equity through action on the social determinants of health: Commission on Social Determinants of Health final report. *World Health Organization*; 2008.
16. Devarajan S, Shah S. Making services work for India's poor. *Economic and Political Weekly*. 2004 Feb 28:907-19.
17. Fairlie RW. An extension of the Blinder-Oaxaca decomposition technique to logit and probit models. *Journal of economic and social measurement*. 2005 Jan 1;30(4):305-16.
18. Gupta A, Fledderjohann J, Reddy H, Raman VR, Stuckler D, Vellakkal S. Barriers and prospects of India’s conditional cash transfer program to promote institutional delivery care: a qualitative analysis of the supply-side perspectives. *BMC health services research*. 2018 Dec;18(1):40.
19. Gwatkin DR. Health inequalities and the health of the poor: what do we know? What can we do?. *Bulletin of the world health organization*. 2000;78:3-18.
20. Gwatkin DR, Bhuiya A, Victora CG. Making health systems more equitable. *The Lancet*. 2004 Oct 2;364(9441):1273-80.
21. Houweling TA, Ronsmans C, Campbell OM, Kunst AE. Huge poor-rich inequalities in maternity care: an international comparative study of maternity and child care in developing countries. *Bulletin of the World Health Organization*. 2007;85:745-54.
22. Hunter BM, Bisht R, Chakravarthi I, Murray SF. Demand-side financing and promotion of maternal health: what has India learnt?. *Economic and Political Weekly*. 2014 Jan 11:66-73.
23. India, U. N. F. P. A. Concurrent assessment of Janani Suraksha Yojana (JSY) in selected states *UNFPA* 2009.
24. Jarris P, Savage-Narva Y, Lupi MV. Promoting health equity and optimal health for all. *Journal of Public Health Management and Practice*, *2016.* 22, S5–S7.
25. Jeffery P, Jeffery R. Only when the boat has started sinking: a maternal death in rural north India. *Social Science & Medicine*. 2010 Nov 1;71(10):1711-8. <https://doi.org/10.1016/j.socscimed.2010.05.002>
26. Kulkarni, P. M.&Baraik, V. K. Utilisation of healthcare services by Scheduled Castes in India (*Working Paper IIDS).* New Delhi: National Family Health Survey 2003.
27. Lagarde M, Haines A, Palmer N. Conditional cash transfers for improving uptake of health interventions in low-and middle-income countries: a systematic review. *JAMA*. 2007 Oct 24;298(16):1900-10.
28. Lim SS, Dandona L, Hoisington JA, James SL, Hogan MC, Gakidou E. India's Janani Suraksha Yojana, a conditional cash transfer programme to increase births in health facilities: an impact evaluation. *The Lancet*. 2010 Jun 5;375(9730):2009-23.
29. Marmot M. Social determinants of health inequalities. *The Lancet*. 2005 Mar 19;365(9464):1099-104.
30. Marmot M, Allen JJ. Social determinants of health equity. 2014:104 Suppl4, S517–S519. DOI:10.2105/*AJPH*.2014.302200.
31. Carvalho N, Rokicki S. The impact of India’s Janani Suraksha Yojana conditional cash transfer programme: A replication study. *The Journal of Development Studies*. 2019 May 4;55(5):989-1006.
32. Navarro V, Muntaner C, Borrell C, Benach J, Quiroga Á, Rodríguez-Sanz M, Vergés N, Pasarín MI. Politics and health outcomes. *The Lancet*. 2006 Sep 16;368(9540):1033-7.
33. Nayar KR. Social exclusion, caste & health: a review based on the social determinants framework. *Indian Journal of Medical Research*. 2007 Oct 1;126(4):355.
34. Navarro V, Shi L. The political context of social inequalities and health. *International Journal of Health Services*. 2001 Jan;31(1):1-21.
35. Oaxaca R. Male-female wage differentials in urban labor markets. *International economic review*. 1973 Oct 1:693-709.
36. Powell-Jackson T, Mazumdar S, Mills A. Financial incentives in health: New evidence from India's Janani Suraksha Yojana. *Journal of health economics*. 2015 Sep 1;43:154-69. <https://doi.org/10.1016/j.jhealeco.2015.07.001>
37. Purnell TS, Calhoun EA, Golden SH, Halladay JR, Krok-Schoen JL, Appelhans BM, Cooper LA. Achieving health equity: closing the gaps in health care disparities, interventions, and research. *Health Affairs*. 2016 Aug 1;35(8):1410-5. <https://doi.org/10.1377/hlthaff.2016.0158>
38. Randive B, San Sebastian M, De Costa A, Lindholm L. Inequalities in institutional delivery uptake and maternal mortality reduction in the context of cash incentive program, Janani Suraksha Yojana: results from nine states in India. *Social Science & Medicine*. 2014 Dec 1;123:1-6.
39. Sabharwal NS. Caste, religion and malnutrition linkages. *Economic and Political Weekly*. 2011 Dec 10:16-18.
40. UNICEF, India. Coverage Evaluation Survey (CES), 2009: All India report. New Delhi. 2010.
41. Vellakkal S, Reddy H, Gupta A, Chandran A, Fledderjohann J, Stuckler D. A qualitative study of factors impacting accessing of institutional delivery care in the context of India's cash incentive program. *Social Science & Medicine*. 2017 Apr 1;178:55-65.
42. Thorat S, Attewell P. The legacy of social exclusion: A correspondence study of job discrimination in India. *Economic and political weekly*. 2007 Oct 13:4141-5.
43. V Karthick, and Madheswaran S. Access to Formal Credit in the Indian Agriculture: Does Caste matter? *Journal of Social Inclusion Studies*, 2018 *4*(2), 169–195. <https://doi.org/10.1177/2394481118814064>
44. Ito T. Caste discrimination and transaction costs in the labor market: Evidence from rural North India. Journal of development Economics. 2009 Mar 1;88(2):292-300.
45. Prakash A. Dalit Capital-State, Markets and Civil Society in Urban India. *Taylor & Francis Limited*; 2014.
46. Kijima Y. Caste and tribe inequality: evidence from India, 1983–1999. *Economic Development and Cultural Change*. 2006 Jan;54(2):369-404.
47. Deshpande A. Does caste still define disparity? A look at inequality in Kerala, India. American Economic Review. 2000 May;90(2):322-5.
48. Ministry of Health and Family Welfare (MOHFW). National Rural Health Mission (2005–2012), Mission Document.
49. Khubchandani J, Soni A, Fahey N, Raithatha N, Prabhakaran A, Byatt N, Simas TA, Phatak A, Rosal M, Nimbalkar S, Allison JJ. Caste matters: perceived discrimination among women in rural India. *Archives of women's mental health*. 2018 Apr 1;21(2):163-70.
50. Vellakkal S, Gupta A, Khan Z, Stuckler D, Reeves A, Ebrahim S, Bowling A, Doyle P. Has India’s national rural health mission reduced inequities in maternal health services? A pre-post repeated cross-sectional study. *Health Policy and Planning*. 2017 Feb 1;32(1):79-90.
51. Sanneving L, Trygg N, Saxena D, Mavalankar D, Thomsen S. Inequity in India: the case of maternal and reproductive health. Global health action. 2013 Dec 1;6(1):19145. 1-31
52. Mishra PS, Syamala T. Multiple Vulnerabilities in Utilising Maternal and Child Health Services in Uttar Pradesh, India. Econ Polit Wkly. 2020;55(43):45–52.
53. Mohanty SK, Srivastava A. Out-of-pocket expenditure on institutional delivery in India. Health policy and planning. 2013 May 1;28(3):247-62.
54. Prinja S, Bahuguna P, Gupta R, Sharma A, Rana SK, Kumar R. Coverage and financial risk protection for institutional delivery: how universal is provision of maternal health care in India?. PloS one. 2015 Sep 8;10(9):e0137315.
55. Mishra S, Mohanty SK. Out-of-pocket expenditure and distress financing on institutional delivery in India. International journal for equity in health. 2019 Dec;18(1):99.
56. Mohanty SK, Kastor A. Out-of-pocket expenditure and catastrophic health spending on maternal care in public and private health centres in India: a comparative study of pre and post national health mission period. Health Economics Review. 2017 Dec 1;7(1):31.
57. Saroha E, Altarac M, Sibley LM. Caste and maternal health care service use among rural Hindu women in Maitha, Uttar Pradesh, India. Journal of midwifery & women's health. 2008 Sep 1;53(5):e41-7.
